# Intracerebroventricular enzyme replacement therapy with β-galactosidase reverses brain pathologies due to GM1 gangliosidosis in mice

**DOI:** 10.1074/jbc.RA119.009811

**Published:** 2019-09-03

**Authors:** Joseph C. Chen, Amanda R. Luu, Nathan Wise, Rolando De Angelis, Vishal Agrawal, Linley Mangini, Jon Vincelette, Britta Handyside, Harry Sterling, Melanie J. Lo, Hio Wong, Nicole Galicia, Glenn Pacheco, Jeremy Van Vleet, Alexander Giaramita, Sylvia Fong, Sushmita M. Roy, Chuck Hague, Roger Lawrence, Sherry Bullens, Terri M. Christianson, Alessandra d'Azzo, Brett E. Crawford, Stuart Bunting, Jonathan H. LeBowitz, Gouri Yogalingam

**Affiliations:** ‡ Research, BioMarin Pharmaceutical, Inc., Novato, California 94949; § Process Sciences, BioMarin Pharmaceutical, Inc., Novato, California 94949; ¶ Department of Genetics, St. Jude Children's Research Hospital, Memphis, Tennessee 38105

**Keywords:** lysosomal storage disease, gene therapy, unfolded protein response (UPR), toxicity, biophysics, beta-galactosidase, cation-independent mannose-6-phosphate receptor, enzyme replacement therapy (ERT), lysosome, neurodegeneration, endoplasmic reticulum stress, safety, GM1 gangliosidosis

## Abstract

Autosomal recessive mutations in the galactosidase β1 (*GLB1*) gene cause lysosomal β-gal deficiency, resulting in accumulation of galactose-containing substrates and onset of the progressive and fatal neurodegenerative lysosomal storage disease, GM1 gangliosidosis. Here, an enzyme replacement therapy (ERT) approach in fibroblasts from GM1 gangliosidosis patients with recombinant human β-gal (rhβ-gal) produced in Chinese hamster ovary cells enabled direct and precise rhβ-gal delivery to acidified lysosomes. A single, low dose (3 nm) of rhβ-gal was sufficient for normalizing β-gal activity and mediating substrate clearance for several weeks. We found that rhβ-gal uptake by the fibroblasts is dose-dependent and saturable and can be competitively inhibited by mannose 6-phosphate, suggesting cation-independent, mannose 6-phosphate receptor–mediated endocytosis from the cell surface. A single intracerebroventricularly (ICV) administered dose of rhβ-gal (100 μg) resulted in broad bilateral biodistribution of rhβ-gal to critical regions of pathology in a mouse model of GM1 gangliosidosis. Weekly ICV dosing of rhβ-gal for 8 weeks substantially reduced brain levels of ganglioside and oligosaccharide substrates and reversed well-established secondary neuropathology. Of note, unlike with the ERT approach, chronic lentivirus-mediated GLB1 overexpression in the GM1 gangliosidosis patient fibroblasts caused accumulation of a prelysosomal pool of β-gal, resulting in activation of the unfolded protein response and endoplasmic reticulum stress. This outcome was unsurprising in light of our *in vitro* biophysical findings for rhβ-gal, which include pH-dependent and concentration-dependent stability and dynamic self-association. Collectively, our results highlight that ICV-ERT is an effective therapeutic intervention for managing GM1 gangliosidosis potentially more safely than with gene therapy approaches.

GM1^3^ gangliosidosis exhibits an autosomal recessive mode of inheritance and arises from mutations in the *GLB1* gene (1). GLB1 encodes lysosomal β-gal, which catalyzes the stepwise lysosomal degradation of multiple galactose-containing substrates (2). Three pathogenic and biochemically distinct classes of galactose-containing substrates accumulate in lysosomes of β-gal–deficient cells; GM1 and GA1 gangliosides, the glycosaminoglycan, keratan sulfate (KS), and oligosaccharides derived from glycoprotein metabolism (glycan substrates) (3, 4, 39). A wide spectrum of clinical phenotypes have been described for GM1 gangliosidosis. In its severest form, infantile-onset GM1 gangliosidosis patients exhibit developmental delay within the first year of life, which coincides with very low levels of residual mutant β-gal activity, accumulation of GM1 ganglioside predominantly in neurons of the brain, and widespread CNS degeneration (2). These patients rapidly lose all motor skills, with death occurring by 2–4.5 years of age (1). GM1 gangliosidosis patients with higher levels of residual mutant β-gal activity present with late-infantile, juvenile, and adult-onset forms of CNS-related disease progression, with a longer survival (5, 6). GM1 gangliosidosis patients also manifest several additional symptoms due to substrate accumulation in various systemic tissues, which coincide with less life-threatening but severely debilitating symptoms of the skeletal system, including epiphyseal dysplasia, scoliosis, and hip dysplasia (5).

The entire range of clinical phenotypes for GM1 gangliosidosis, ranging from severe to relatively mild are clustered within a narrow range of residual lysosomal β-gal activity, from 0 to 15% of normal control levels (7, 8), suggesting that therapeutic strategies for this disease do not necessarily need to completely normalize β-gal activity to mediate lysosomal degradation of stored substrates and prevent disease progression. In support of this, we have previously demonstrated in patient cells that only very small increases in residual enzyme activity (∼20% of normal enzyme activity levels) are sufficient to completely clear lysosomal storage material in a related lysosomal storage disease, Sanfilippo B syndrome (9).

Many approved enzyme replacement therapy (ERT) regimes for nonneurodegenerative lysosomal storage diseases have exploited the cell surface cation-independent mannose 6-phosphate receptor (CI-MPR) targeting pathway, where mannose 6-phosphorylated glycans present on the ERT enzyme bind avidly to the CI-MPR, resulting in their internalization into clathrin-coated vesicles and delivery to lysosomes of patient cells (10, 11). More recently, intermittent intracerebroventricular (ICV)-administered ERT has been investigated to overcome the blood–brain barrier obstacle and directly deliver lysosomal enzymes into the ventricles of the brain, with cellular uptake of enzyme coinciding with substrate clearance and reversal of several aspects of well-entrenched secondary neuropathology in animal models of neurodegenerative lysosomal storage disease (12–14).

In recent years, preclinical AAV-mediated gene therapy studies have been investigated for GM1 gangliosidosis by several academic groups, with evidence of effective substrate turnover in the brain and increased survival in mouse and feline models of the disease (15–17). AAV-mediated gene therapy for GM1 gangliosidosis patients has also been pursued, with the first patient recently intravenously dosed in an ongoing clinical program sponsored by Axovant Sciences Ltd.^4^ Preclinical studies for CNS-directed AAV-mediated gene therapy are also currently being pursued by LysoGene and PassageBio. However, a potential obstacle for developing effective gene therapy for GM1 gangliosidosis is the propensity for endogenous mutant β-gal (Fig. 9*A*) and overexpressed WT β-gal (Fig. 9*B*) to mislocalize and accumulate in the endoplasmic reticulum (ER) (18). Evidence of eosinophilic granules in neurons of a feline model of GM1 gangliosidosis treated with AAV-mediated GLB1 gene therapy has also been reported (17). These observations warrant investigating potential toxicity associated with developing therapies to augment β-gal levels in GM1 gangliosidosis patients.

Here, we evaluated an ERT approach to circumvent the ER and directly target purified recombinant human β-gal (rhβ-gal) to lysosomes of GM1 gangliosidosis patient fibroblasts and in a mouse model of the disease via cell surface receptor–mediated endocytosis. We also employed a lentivirus-mediated, CMV promoter–driven gene therapy approach to overexpress GLB1 in GM1 gangliosidosis patient fibroblasts and further understand how retention of overexpressed β-gal in the ER impacts on cellular stress. We then evaluated the biophysical properties of rhβ-gal at neutral and acidic pH to predict how this glycosidase is affected when retained in the pH neutral environment of the ER and in acidified lysosomes following β-gal augmentation therapy.

## Results

### A single low-nanomolar dose of purified rhβ-gal exhibits highly efficient CI-MPR–mediated cellular uptake in patient cells, which coincides with substrate clearance for several weeks

**Figure 1.**
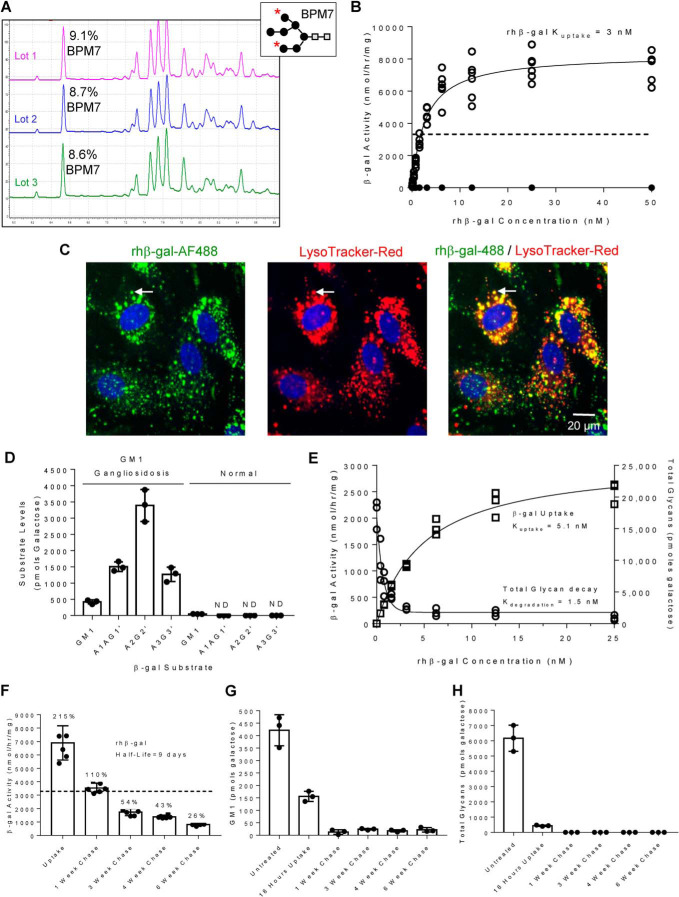
**Purified rhβ-gal exhibits highly efficient CI-MPR–mediated cellular uptake in GM1 gangliosidosis patient fibroblasts, which coincides with substrate clearance for up to 6 weeks.** *A*, oligosaccharide analysis of three independent production and purification campaigns for rhβ-gal (lots 1, 2, and 3) by CZE. The indicated BPM7 peak ID is assigned from co-migration with a known reference lysosomal enzyme. *Inset*, BPM7 structure; mannose indicated with *black circles* and GlcNAc indicated with *gray squares*. *, two phosphorylated mannose sites on BPM7. *B*, representative *K*_uptake_ determination experiment for rhβ-gal cellular uptake in GM1 gangliosidosis patient fibroblasts (GM05653). Six independent cultures of cells were incubated with increasing concentrations of rhβ-gal for 24 h in the absence (*open circles*) or presence (*closed circles*) of 8 mm M6P. Uptakes with lower concentrations of M6P were also tested and are included in Fig. S1. The *dashed line* represents the level of β-gal activity detected in normal fibroblasts. Average *K*_uptake_ = 3.4 ± 1.1 nm, *n* = 13; average *V*_max_ = 7388 ± 1513 nmol/h/mg, *n* = 13. *C*, representative images of paraformaldehyde-fixed GM1 gangliosidosis patient fibroblasts (GM05653) following a 24-h incubation with 25 nm Alexa Fluor 488 β-gal (*green channel*). Prior to fixation, cells were incubated with LysoTracker Red (*red channel*). An example of β-gal co-localization with a LysoTracker-Red^+^ acidified organelle is indicated with an *arrow. D*, comparative substrate profiling by CZE of GM1 ganglioside and multiple glycan substrates detected in GM1 gangliosidosis patient fibroblasts (GM03589), which are negligible or absent in fibroblasts from a normal individual. The structure of A1G1′, A2G2′, and A3G3′ and their individual *K*_degradation_ rates are shown in Fig. S2. *E*, *K*_degradation_ determination for rhβ-gal-mediated clearance of the three major glycan substrates that accumulate in GM1 gangliosidosis patient fibroblasts (GM03589). Triplicate cultures of cells were incubated for 4 h with increasing doses of rhβ-gal. The uptake medium was removed, and cells were washed and chased for a further 6 h in the absence of enzyme. Cells lysates were prepared and then assayed for β-gal activity (*squares*) or total glycans (A1G1′ + A2G2′ + A3G3′; *circles*). *n* = 2 repeats. *F–H*, representative rhβ-gal half-life determination experiment in GM1 gangliosidosis patient fibroblasts (GM05653; *F*) and correlation with substrate levels. Triplicate cultures of cells were grown for 10 days to permit substrate accumulation and then incubated with 3 nm rhβ-gal for 18 h, at which time the enzyme was removed and cells were maintained in growth medium for up to 6 weeks. At each time point indicated, β-gal activity (*F*), GM1 ganglioside (*G*), or total glycan levels (A1G1′ + A2G2′ + A3G3′; *H*) were measured. *n* = 2 repeats. *Error bars* represent the standard deviation (S.D.).

Five consensus *N*-linked glycosylation sites have previously been reported on β-gal produced in Chinese hamster ovary (CHO) cells (19). *N*-Linked oligosaccharide profiling of peptide:*N*-glycosidase F–digested glycans from three individual production and purification runs of rhβ-gal by capillary zone electrophoresis (CZE) suggest that ∼9% of the total glycan composition of rβ-gal is bisphosphorylated oligomannose (BPM7; Fig. 1*A*), the preferred glycan moiety for CI-MPR–dependent cellular uptake and lysosomal targeting (10). The purified material from lot 3 was used for all further results generated in this work. In GM1 gangliosidosis patient fibroblasts, very low-nanomolar doses of rhβ-gal cellular uptake over 24 h are sufficient to normalize β-gal activity levels (Fig. 1*B*). Cellular uptake with rhβ-gal can be completely abolished with the addition of 8 mm mannose 6-phosphate (M6P), a known inhibitor of CI-MPR–mediated cellular enzyme uptake (Fig. 1*B*). Lower doses of M6P, down to 1 mm, are also sufficient to prevent β-gal uptake (Fig. S1). *K*_uptake_, defined as the concentration of enzyme at half-maximal cellular uptake, is 3.4 ± 1.1 nm (*n* = 13 repeats; Fig. 1*B*), with a maximal uptake capacity (*V*_max_) corresponding to 7338 ± 1513 nmol/h/mg (Fig. 1*B*). Following cellular uptake, rhβ-gal co-localizes with LysoTracker Red, a marker of acidified lysosomes, suggestive of successful delivery of enzyme to lysosomes (Fig. 1*C*). Comparative substrate profiling of GM1 gangliosidosis patient fibroblasts by CZE reveals the accumulation of a series of glycan substrates (A1G1′, A2G2′, and A3G3′; Fig. 1*D*, with structures shown in Fig. S2). These glycan substrates can be rapidly cleared following a 4-h exposure to very low-nanomolar concentrations of purified rhβ-gal, with half-maximal clearance (*K*_degradation_) being achieved at 1.5 nm, well below the *K*_uptake_ (5.1 nm; Fig. 1*E*, with individual *K*_degradation_ rates for A1G1′, A2G2′, and A3G3′ shown in Fig. S2). In addition to the glycan substrates, GM1 ganglioside substrate was also readily detected by CZE in skin fibroblasts from a GM1 gangliosidosis patient fibroblasts by CZE (Fig. 1*D*) and by high-content imaging with a GM1 polyclonal antibody (Fig. S3). Following cellular uptake of a single low dose of rhβ-gal (3 nm) for 18 h, lysosome-delivered rhβ-gal activity decays slowly with a half-life of ∼9 days in GM1 gangliosidosis patient fibroblasts (Fig. 1*F*), which coincides with rapid turnover of GM1 ganglioside substrate (Fig. 1*G* and Fig. S3) and glycan substrates (Fig. 1*H*) within 1 week. Furthermore, a single low dose (3 nm) of rhβ-gal delivered to lysosomes of GM1 gangliosidosis patient fibroblasts is sufficient to prevent reaccumulation of GM1 ganglioside substrate (Fig. 1*G* and Fig. S3) and glycan substrates (Fig. 1*H*) for up to 6 weeks.

### ICV-ERT with rhβ-gal augments lysosomal β-gal activity in hippocampal neurons of the GM1 gangliosidosis mouse brain

**Figure 2.**
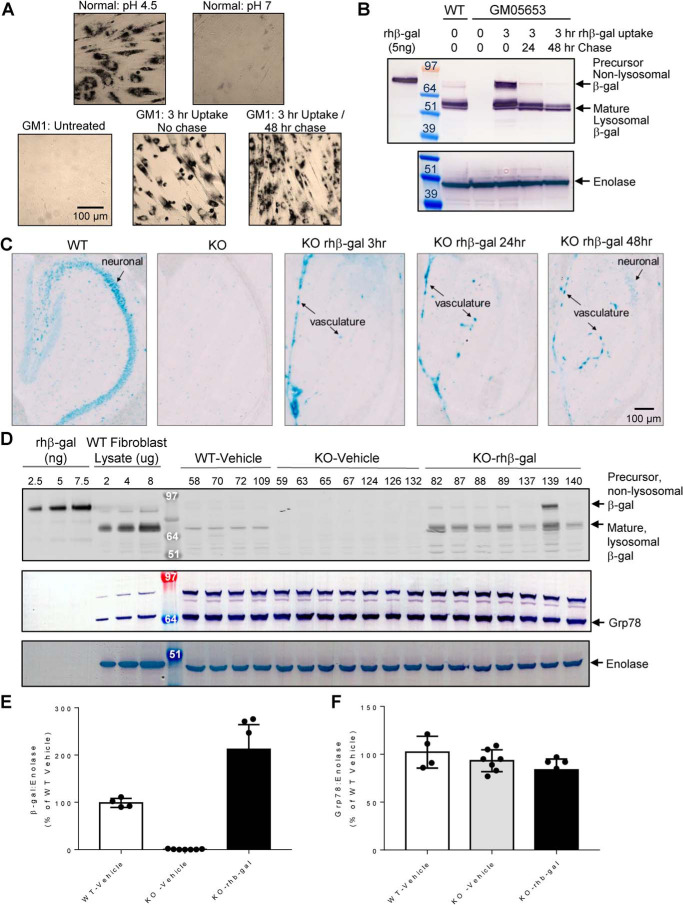
**ICV-ERT with rhβ-gal augments lysosomal β-gal activity in patient fibroblasts and hippocampal neurons of the GM1 gangliosidosis mouse brain.** *A*, *in situ* detection at ×40 magnification of β-gal activity observed in normal fibroblasts at pH 4.5 and 7 after 18 h using X-gal substrate. X-gal staining was also performed in control GM1 gangliosidosis untreated fibroblasts (GM05653) or following 3 h of cellular uptake with 100 nm rhβ-gal. Alternatively, after cellular uptake, cells were washed and chased in growth medium without enzyme for a further 48 h. *B*, β-gal Western blotting of cells described in *A*. Enolase was used as a loading control. *C*, *in situ* detection, at ×20 magnification, of β-gal activity at acidic pH using X-gal substrate in cryo-frozen sagittal brain sections from a 16-week-old control WT mice, a control GLB1 KO mouse, or GLB1 KO mice following a single ICV dose (100 μg) of rhβ-gal. rhβ-gal ICV-ERT–treated mice were taken down 3, 24, or 48 h after ICV administration. *Blue signal* indicates areas of β-gal activity. *Black arrows* indicate development of signal in the CA3 region of the hippocampus (neuronal) and in vascular structures. *D*, Western blotting of brain homogenates (*left* and *right* hemispheres) prepared from individual WT-vehicle (*n* = 4), KO-vehicle (*n* = 7), or KO-rhβ-gal (*n* = 7) mice after receiving four ICV doses of rhβ-gal (100 μg/dose) over 2 weeks, with mice being taken down 24 h after the last dose. Mouse numbers are internal mouse ID numbers assigned by the GM1 mouse study subgroup. For comparison, 2.5, 5, or 7.5 ng of rhβ-gal was also included on gels as an indicator of the precursor, nonlysosomal enzyme. 2, 4, or 8 μg of fibroblast lysate prepared from normal human fibroblasts was used as an indicator of mature, lysosomal β-gal. *E*, quantification of mature β-gal protein levels shown in *D*, standardized to the enolase loading control and expressed as individual values, *error bars* represent the standard deviation (S.D.). *F*, quantification of Grp78 protein levels shown in *D*, standardized to the enolase loading control and expressed as individual values with the S.D. indicated.

When rhβ-gal is delivered to the acidified lumen of the lysosome, proteolytic cleavage generates mature β-gal, resulting in a ∼20-kDa reduction in mass (20), which can be monitored by Western blotting as an indicator of successful β-gal delivery to lysosomes. X-gal substrate, which is specifically cleaved by β-gal under acidic conditions, can also be used to detect β-gal activity *in situ*. We initially tested both of these methods in GM1 gangliosidosis patient fibroblasts to monitor the delivery of rhβ-gal to lysosomes. β-Gal activity toward X-gal substrate in normal human fibroblasts is readily detected *in situ* under acidic conditions but not neutral pH conditions (Fig. 2*A*). Furthermore, as expected, β-gal protein is predominantly detected as the mature, lysosomal enzyme in normal fibroblasts by Western blotting (Fig. 2*B*). β-Gal activity toward X-gal substrate is not detected *in situ* in GM1 gangliosidosis patient fibroblasts but is readily detected following a short 3-h exposure to rhβ-gal (Fig. 2*A*), which coincides with the appearance of mature, lysosomal rhβ-gal as well as the precursor nonlysosomal enzyme by Western blotting (Fig. 2*B*), suggestive of partial delivery of rhβ-gal to lysosomes. Following enzyme withdrawal and a 48-h chase, β-gal activity toward X-gal substrate *in situ* remains detectable (Fig. 2*A*), with the majority of internalized enzyme by this time point corresponding to mature lysosomal rhβ-gal by Western blotting (Fig. 2*B*).

To determine the time required for ICV administered rhβ-gal to reach the lysosomes of neurons in the GM1 gangliosidosis mouse brain (21), we followed a single 100-μg ICV dose of rhβ-gal and evaluated β-gal activity toward X-gal substrate at lysosomal pH *in situ* over a 48-h chase period. In frozen sagittal brain sections, there are strong patterns of cellular neuron-like X-gal signal in the WT hippocampus (Fig. 2*C*), a critical region of GM1 gangliosidosis disease pathogenesis. *In situ* detection of β-gal activity in normal hippocampus mouse neurons also coincides with the detection of mature, lysosomal endogenous β-gal in normal mouse brain homogenates by Western blotting (Fig. 2*D*; quantified in Fig. 2*E*). In contrast, β-gal activity toward X-gal substrate is completely absent in the hippocampus neurons of GLB1 KO mice (Fig. 2*C*), along with β-gal protein, as detected by Western blotting (Fig. 2*D*; quantified in Fig. 2*E*). Following an ICV administered dose (100 μg) of rhβ-gal, β-gal activity toward X-gal substrate in hippocampal neurons is not initially detected 3 or 24 h after enzyme administration but becomes apparent after 48 h (Fig. 2*C*). These results suggest that ICV mediated delivery of enzymatically active rhβ-gal to lysosomes of neurons in the GM1 gangliosidosis mouse brain requires several days and most likely will require an intermittent ICV-ERT dosing strategy. In support of this, four ICV-ERT injections with rhβ-gal over 2 weeks result in the detection of mature, lysosomal β-gal in brain homogenates of GLB1 KO mice (Fig. 2*D*; quantified in Fig. 2*E*), with only one of seven treated GLB1 KO mice showing elevated levels of prelysosomal rhβ-gal enzyme (Fig. 2*D*, mouse 139).

### ICV administered rhβ-gal exhibits broad bilateral bio-distribution throughout the brain, which coincides with clearance of multiple substrates in GM1 gangliosidosis mice

**Figure 3.**
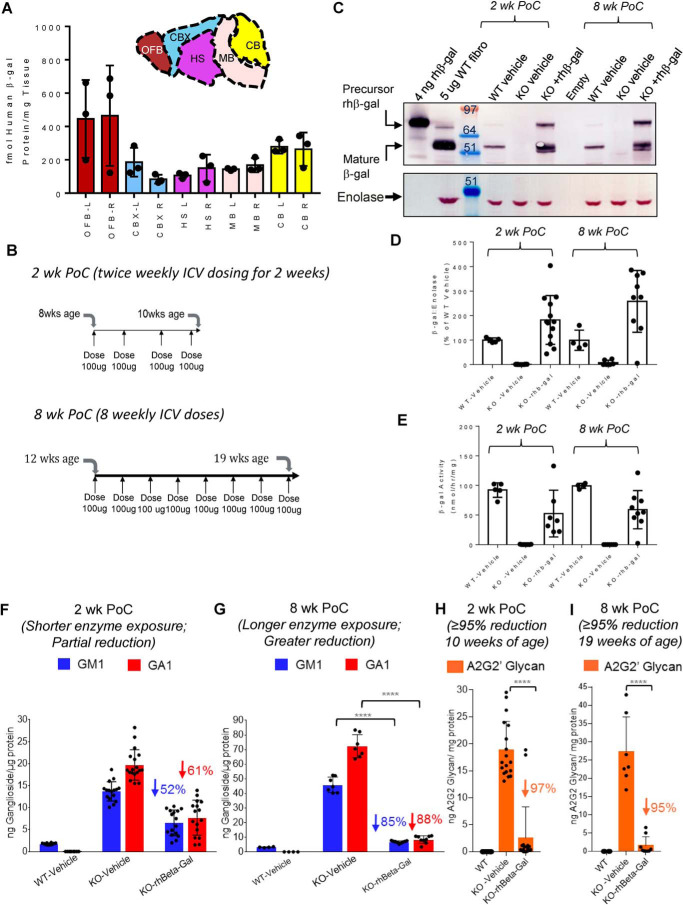
**ICV administered rhβ-gal exhibits broad bilateral bio-distribution throughout the brain, which coincides with clearance of multiple substrates in GM1 gangliosidosis mice.** *A*, detection of rhβ-gal in WT mouse brain tissue (*n* = 3 mice) 3 h after a single, unilateral injection (100-μg dose) into the lateral ventricle utilizing MS. *Colored bars* represent detection of enzyme activity in each macro-dissected area. Mouse left and right brain hemispheres were each dissected into the following regions: OFB, CBX, HS, CB, and MB (containing pons and medulla) and analyzed individually. Each *pair* of *bars* represents signal in the left (*L*) or right (*R*) sagittal hemispheres. *B*, summary of the 2-week and 8-week short-term proof-of-concept (*2 wk* or *8 wk PoC*) ICV-ERT studies evaluated in the GM1 gangliosidosis mouse model. The 2-week PoC study commenced at 8 weeks of age, with mice receiving four ICV doses (100 μg/dose) of rhβ-gal or vehicle (aCSF) over 2 weeks. The 8-week PoC study commenced at 12 weeks of age, with mice receiving weekly ICV dosing (100 μg/dose) with rhβ-gal or vehicle for 8 weeks. Mice were taken down 24 h after the final ICV dose of enzyme. *C*, representative β-gal Western blotting of β-gal protein levels in pooled brain homogenates prepared from the left brain hemisphere of WT or GLB1 KO mice treated with vehicle or rhβ-gal, as summarized in *B*. For comparison, 4 ng of purified rhβ-gal was also included on gels as an indicator of the precursor, nonlysosomal enzyme. Also included was 5 μg of cell lysate prepared from WT human fibroblasts (*WT fibro*) as an indicator of mature β-gal successfully delivered to lysosomes. *D*, quantification of mature lysosomal β-gal protein levels in brain homogenates from Western blots of individual samples, standardized to an enolase loading control of Western blots described in *C*. Quantified results are expressed as individual values, *error bars* represent the standard deviation (S.D.). *2 wk PoC*, WT vehicle, *n* = 5; KO vehicle, *n* = 6; KO-rhβ-gal, *n* = 7. *8 wk PoC*, WT vehicle, *n* = 4; KO vehicle *n* = 7; KO rhβ-gal, *n* = 9). *E*, corresponding β-gal activity levels detected in brain homogenates prepared from mice, with results expressed as individual values with the S.D. indicated. *2 wk PoC*, WT vehicle, *n* = 5; KO vehicle, *n* = 9; KO rhβ-gal, *n* = 7. *8 wk PoC*, WT vehicle, *n* = 4; KO vehicle, *n* = 6; KO rhβ-gal, *n* = 9. *F–I*, GM1 and GA1 ganglioside levels (*F* and *G*) and A2G2′ glycan levels (*H* and *I*) detected in brain homogenates from the 2-week PoC (WT vehicle, *n* = 5; KO vehicle, *n* = 9; KO-rhβ-gal, *n* = 7) and 8-week PoC mice (WT vehicle, *n* = 4; KO vehicle, *n* = 7; KO rhβ-gal, *n* = 9). ****, *p* < 0.0001.

A single 100-μg ICV dose of rhβ-gal into WT mice coincides with broad bilateral distribution of the enzyme throughout the brain 24 h after administration, as determined using a MS-based assay (Fig. 3*A*). Two short-term proof-of-concept (PoC) ICV-ERT dosing experiments were evaluated, commencing at 8 weeks of age or at 12 weeks of age (Fig. 3*B*), well after secondary neuroinflammation has commenced in this mouse model of GM1 gangliosidosis (22–25). Twice weekly ICV dosing in GLB1 KO mice over 2 weeks (*2 wk PoC*; Fig. 3*B*) or weekly ICV dosing for 8 weeks (*8 wk PoC*; Fig. 3*B*) results in the detection of mature lysosomal rhβ-gal protein by Western blotting in brain homogenates (Fig. 3*C*; see Fig. 3*D* for quantification), which coincides with normalization of β-gal activity to varying extents in individual mice (Fig. 3*E*). Weekly ICV dosing with rhβ-gal for 8 weeks in GLB1 KO mice also coincides with near-to-complete clearance of two classes of substrates in GM1 gangliosidosis brain tissue, with the GM1 and GA1 ganglioside substrates requiring a longer duration of ERT for maximal clearance, when compared with glycan substrates (Fig. 3, *F–I*).

### ICV administered rhβ-gal reverses secondary neuropathology in GM1 gangliosidosis mice

**Figure 4.**
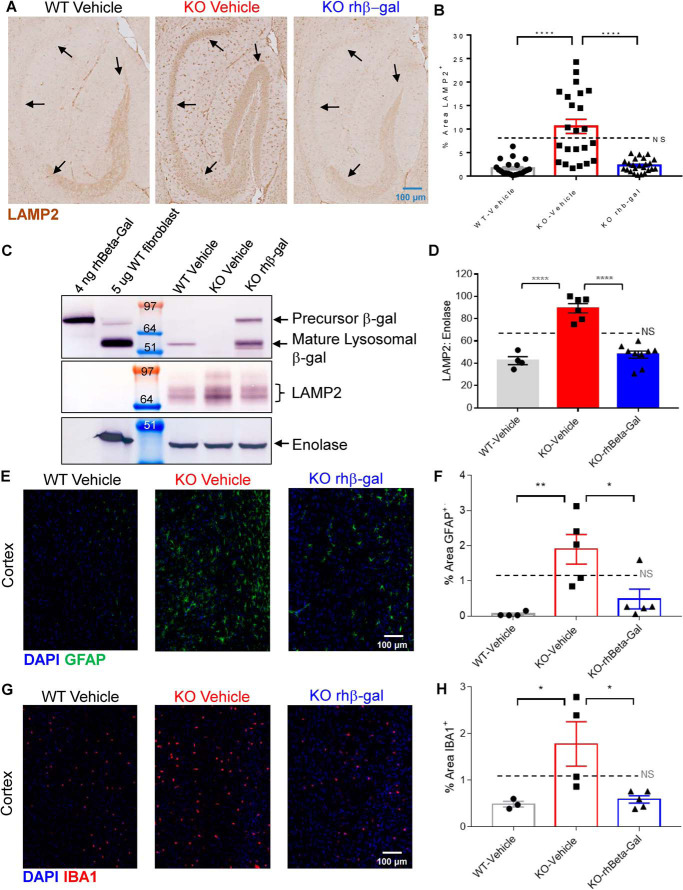
**ICV administered rhβ-gal reverses secondary neuropathology in GM1 gangliosidosis mice.** *A*, representative LAMP2 IHC images (20× magnification) of sagittal hippocampus sections prepared from the left brain hemisphere from mice dosed weekly for 8 weeks (dosing regimen indicated in Fig. 3*B*). *Black arrows*, presence (KO vehicle) or absence (WT vehicle and KO rhβ-gal-treated) of LAMP2 signal in the CA1–CA3 and hippocampal neurons. *B*, quantitative analysis of the percentage of area of LAMP2-positive signal measured over the cortex, hippocampus, thalamus, brain stem and cerebellum. *n* = 4–6 mice for each group. *C*, representative β-gal, LAMP2, and enolase Western blots of pooled brain homogenates prepared from the left hemispheres from WT or GLB1 KO mice treated with eight weekly doses of vehicle or rhβ-gal: WT vehicle, *n* = 4; KO vehicle, *n* = 6; KO rhβ-gal, *n* = 9. *D*, quantification of LAMP-2 protein levels from Western blots of individual samples, standardized to an enolase loading control, with individual values indicated *error bars* represent the standard deviation (S.D.). WT vehicle, *n* = 4; KO vehicle, *n* = 6; KO rhβ-gal, *n* = 9. *E–H*, representative GFAP IF (*E*) or IBA1 IF (*G*) images of cortical mouse brain tissue. Corresponding percentage area analyses for cortical GFAP (*F*) or IBA1 (*H*) are indicated. All quantitative data are represented as individual values *error bars* represent the standard error of the mean (SEM). *GFAP IF*, WT vehicle, *n* = 4; KO vehicle, *n* = 5; KO rhβ-gal, *n* = 5. *IBA1 IF*, WT vehicle, *n* = 3; KO vehicle, *n* = 3, KO rhβ-gal, *n* = 5. ****, *p* < 0.0001. **, *p* < 0.01; *, *p* < 0.05.

GLB1 KO mice contain elevated levels of LAMP2 protein, a marker of lysosomal storage pathology, throughout the brain, as detected by immunohistochemistry (IHC) using a polyclonal LAMP2 antibody (Fig. 4*A*; see Fig. 4*B* for quantification) and by Western blotting (Fig. 4*C*; see Fig. 4*D* for quantification), when compared with vehicle-treated WT mice. ICV dosing with rhβ-gal for 8 weeks in GLB1 KO mice coincides with normalization of LAMP2 levels, as detected by IHC (Fig. 4*A*; see Fig. 4*B* for quantification) and by Western blotting (Fig. 4*C*; see Fig. 4*D* for quantification). GFAP, a marker for astrogliosis (Fig. 4*E*; see Fig. 4*F* for quantification), as well as IBA1, a marker of microgliosis (Fig. 4*G*; see Fig. 4*H* for quantification), were significantly elevated in the cortex from vehicle-treated KO mice at 3 months of age, when compared with vehicle-treated WT mice by IHC. Both GFAP levels (Fig. 4*E*; see Fig. 4*F* for quantification) and IBA1 levels (Fig. 4*G*; see Fig. 4*H* for quantification) were normalized in the cortex of KO mice following eight weekly ICV doses of rhβ-gal, when compared with vehicle-treated KO mice. Collectively, our results in Figs. 2–4 suggest that in a mouse model of GM1 gangliosidosis, an intermittent ICV-ERT dosing approach with rhβ-gal results in broad, bilateral biodistribution of the enzyme, which coincides with near-to-complete clearance of multiple substrates in the brain and reversal of well-entrenched secondary neuropathology. While dose-ranging and reaccumulation studies were not evaluated in the mouse model, our proof-of-concept studies in patient cells suggest that a single dose of rhβ-gal is sufficient to augment β-gal levels in lysosomes and mediate substrate clearance for up to 6 weeks (Fig. 1).

### ICV-ERT with rhβ-gal for 8 weeks normalizes β-gal activity in systemic tissues, which coincides with a partial reduction in urinary A2G2′ substrate

**Figure 5.**
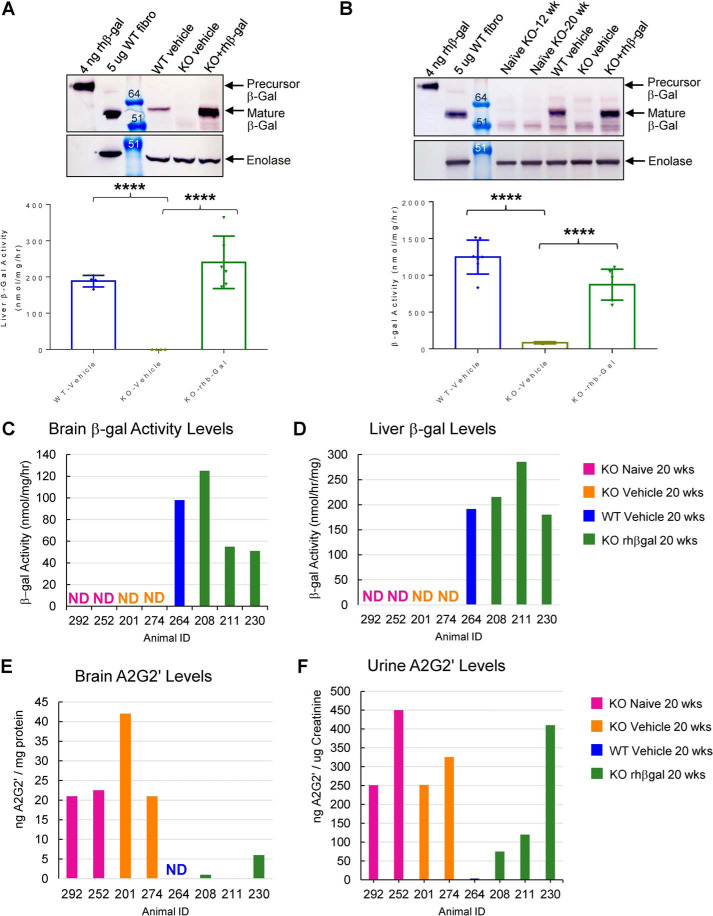
**ICV-ERT with rhβ-gal for 8 weeks normalizes β-gal activity in systemic tissues, which coincides with a partial reduction in urinary A2G2′ substrate.** *A* and *B*, β-gal activity detected in liver tissue (*A*) or bone marrow (*B*) from WT vehicle (*n* = 4–7), KO vehicle (*n* = 6–8), and KO rhβ-gal–treated mice (*n* = 6), with results expressed as individual values *error bars* represent the standard deviation (S.D.). ****, *p* < 0.0001. Also indicated *above* each *graph* is a representative Western blot of β-gal protein levels in pooled liver homogenates (*A*) or bone marrow lysates (*B*) prepared from WT or GLB1 KO mice treated with vehicle or rhβ-gal. For comparison, 4 ng of purified rhβ-gal was also included on gels as an indicator of the precursor, nonlysosomal enzyme. Also included was 5 μg of cell lysate prepared from WT human fibroblasts (*WT fibro*) as an indicator of mature β-gal successfully delivered to lysosomes. Enolase was used as loading control. *C–F*, correlation between β-gal activity levels in brain (*C*) and liver (*D*) with A2G2′ substrate levels detected in brain (*E*) and urine (*F*) from individual mice from the 8-week PoC study. Note that the individual β-gal activity and A2G2′ levels in *C–F* are shown for this small subset of mice from the 8-week PoC study, the reason being that we only managed to successfully collect urine samples from these mice 24 h after the final ICV dose of rhβ-gal. Mouse numbers are internal mouse ID numbers assigned by the GM1 mouse study subgroup.

β-Gal activity toward X-gal substrate was detected *in situ* in GLB1 KO mouse brain tissue immediately following ICV-ERT in areas that appeared vascular-like (Fig. 2*C*), suggesting that ICV delivered enzyme may be gaining access to the systemic circulation. In support of this, eight weekly ICV doses of rhβ-gal in GLB1 KO mice coincides with near-to-complete normalization of β-gal activity levels and β-gal protein levels in the liver (Fig. 5*A*) and bone marrow (Fig. 5*B*) of the majority of animals. Whereas substrate levels in liver and bone marrow of these mice were not measured, the degree of β-gal augmentation in liver and bone marrow is well above the critical threshold of ∼15% of normal residual lysosomal enzyme activity that is needed to mediate substrate turnover and prevent lysosomal storage disease progression in GM1 gangliosidosis patients (7, 8). It is therefore possible that an ICV route of rhβ-gal administration may also result in exposure of enzyme to systemic sites of disease progression in sufficient amounts to mediate substrate clearance.

In our biomarker analyses of GM1 gangliosidosis mice, we observed elevated levels of the A2G2′ glycan substrate in the brain as well as in several systemic tissues, including liver, spleen, kidney and urine, when compared with WT littermate control mice.^4^ A2G2′ glycan substrate was also elevated in brain tissue and urine from GM1 gangliosidosis patients, when compared with samples from normal individuals,^4^ raising the possibility of utilizing urinary A2G2′ levels as an indicator of systemic exposure of rhβ-gal following ICV-ERT. In this study, we only managed to collect urine from a subset of the mice from the 8-week PoC study, 24 h after the final ICV administered dose of rhβ-gal. Naive GLB1 KO mice (*n* = 2), along with vehicle-treated GLB1 KO mice (*n* = 2), contain no detectable β-gal activity in individual brain and liver samples (Fig. 5, *C* and *D*), which coincides with elevated A2G2′ substrate in their corresponding brain and urine samples (Fig. 5, *E* and *F*). In contrast, β-gal activity is readily detected in the brain and liver from a single vehicle-treated WT mouse (Fig. 5, *C* and *D*; *n* = 1), which coincides with no A2G2′ substrate being detected the corresponding brain and urine sample from this mouse (Fig. 5, *E* and *F*). A regimen of intermittent ICV dosing with rhβ-gal for 8 weeks is sufficient to augment β-gal activity to varying levels in individual brain and liver samples from three ICV-ERT–treated GLB1 KO mice (Fig. 5, *C* and *D*), which coincides with clearance of A2G2′ glycan to varying extents in the brain and urine (Fig. 5, *E* and *F*). Therefore, although no conclusion can be made after only eight weekly ICV doses of rhβ-gal in GLB1 KO mice, our biomarker analysis of glycan substrate accumulation in human GM1 gangliosidosis patients,^4^ together with the results in a limited number of animals shown here, warrant further studies to investigate the potential of A2G2′ glycan as a noninvasive biomarker of rhβ-gal systemic exposure and substrate turnover during ICV-ERT clinical trials.

### At low concentrations, β-gal is predominantly a monomer and prone to destabilization at neutral pH and a stable dimer at acidic pH

**Figure 6.**
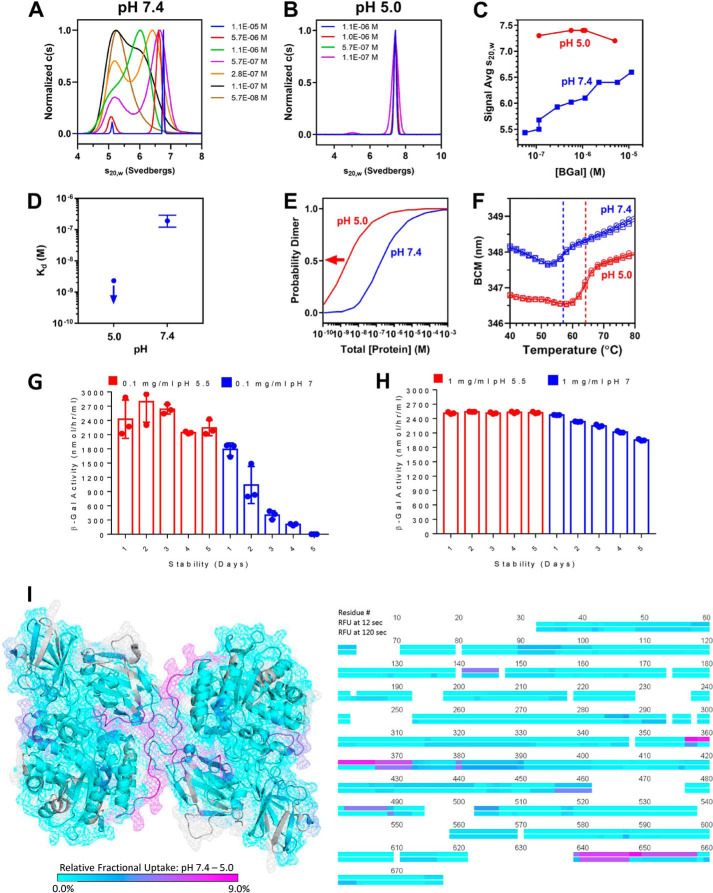
**β-Gal is predominantly a monomer and prone to destabilization at neutral pH and a stable dimer at acidic pH.** *A*, sedimentation coefficient distributions of 0.005–1 mg/ml rhβ-gal at pH 7.4 showing rhβ-gal concentration dependence, where monomer sediments at 5.1 S and dimer at 6.7 S. *B*, sedimentation coefficient distributions of 0.01–0.1 mg/ml rhβ-gal at pH 5.0 showing rhβ-gal concentration independence, where dimer sediments at 6.7 S. *C*, signal average S as a function of total rhβ-gal loading concentration obtained by integrating across the monomer and dimer regions of the distributions in *A* and *B. D*, dimer dissociation constants established by SE-AUC. No evidence of dimer dissociation is observed at pH 5.0, and the *downward arrow* indicates the upper limit of 2 nm at pH 5.0. *E*, probability distribution generated using the equilibrium constants in *D*. Analogous to the *blue arrow* in *D*, because *K_d_* could not be resolved at pH 5.0, the *red arrow* indicates the upper limit for the probability of dimer under these conditions. *F*, differential scanning fluorimetery thermograms at pH 5.0 and 7.4. *BCM*, barycentric mean of the fluorescence emission peak. *G* and *H*, concentration-dependent and pH-dependent stability of rhβ-gal diluted into 1 mm NaPP_i_ at pH 5.5 or 7, at a concentration of 0.1 mg/ml (*G*) or 1 mg/ml (*H*). Stability was determined by measuring activity toward the 4MU β-gal substrate. Results are expressed as individual values. *Error bars* represent the standard deviation (S.D.). *I*, relative fractional uptake (*RFU*) of deuterium (pH 7.4–5.0) after 12 s of deuterium uptake scaled from 0% (*cyan*) to 9% (*magenta*) overlaid on the dimer structure (Protein Data Bank entry 3THC) (27). The same data for 12 and 120 s of deuterium uptake are also shown in linear form (*right*). Gaps in HDX data are shown in *light gray* on the crystal structure and are *blank* in the linear view.

In a previous study, GLB1 overexpression in feline GM1 gangliosidosis fibroblasts has been shown to result in β-gal mislocalization and retention in the ER (18). These observations prompted us to evaluate the biophysical properties of recombinant human β-gal (rhβ-gal) at neutral and acidic pH to predict how this glycosidase is affected when retained in the pH-neutral environment of the ER following β-gal augmentation therapy. The monomer-dimer equilibrium of rhβ-gal was investigated by sedimentation velocity analytical ultracentrifugation (SV-AUC). Results demonstrate that rhβ-gal undergoes dynamic assembly of monomers and dimers at neutral pH (Fig. 6, *A* and *C*) but shows no evidence of monomer-dimer equilibrium at acidic pH (Fig. 6, *B* and *C*). To further characterize the dimerization reaction, sedimentation equilibrium AUC (SE-AUC) experiments were performed under identical buffer conditions (Fig. 6*D*). At neutral pH, the rhβ-gal equilibrium data could not be fit with either a homogenous monomer or dimer model but did fit well to a monomer-dimer equilibrium model with a dissociation constant of 243 nm at 25 °C (68% confidence interval: *K_d_* = 172–381 nm). Consistent with the SV-AUC results, no monomer-dimer equilibrium could be modeled at acidic pH, with only a homogenous dimer observed at all concentrations tested. Because absorbance optics in the AUC are capable of detecting 10% monomer, the *K_d_* at pH 5 was calculated to be no weaker than 2 nm (Fig. 6*D*), which is roughly 100 times stronger than at neutral pH. To illustrate this substantial difference in dimerization constants, probabilities of observing rhβ-gal dimer were calculated as a function of total rhβ-gal concentration (Fig. 6*E*). Apparent from this plot, rhβ-gal dimers are much more likely to form at low-nanomolar rhβ-gal concentrations under acidic conditions, when compared with neutral pH. To evaluate the thermal stability of β-gal under these conditions, differential scanning fluorimetery (DSF) experiments demonstrate that the protein is more stable under conditions that promote dimers (Fig. 6*F*). In support of the DSF results, the stability of rhβ-gal over 5 days at 37 °C was gradually reduced under neutral pH conditions at low concentrations (0.1 mg/ml), when compared with rhβ-gal held under acidic conditions (Fig. 6*G*). This pH-dependent reduction in rhβ-gal stability was less noticeable when rhβ-gal was tested at higher concentrations (1 mg/ml) (Fig. 6*H*).

Hydrogen–deuterium exchange MS (HDX) experiments are in good agreement with the AUC results described above. This is illustrated in Fig. 6*G*, where the relative fractional uptake (pH 7.4 to 5.0) is overlaid on the dimer structure (Protein Data Bank entry 3THC) and is also shown in a linear format. The largest uptake differences at these pH conditions (*magenta*) are in and around the dimer interface proposed by Ohto *et al.* (26) and are consistent with a small monomer population at neutral pH, whereas most of the rest of the structure is unchanged (*cyan*; Fig. 6*I*). These HDX results also provide valuable information about the residues involved in the solution-phase dimer interface. Specifically, the crystal coordinates for residues 648–677 were not determined, presumably due to the limited proteolysis treatment and/or an unstructured C terminus in the pH 8.0 crystallization buffer (26), whereas our HDX data suggest that in an acidic solution, residues ∼630–658 make up a substantial portion of the dimer interface. In addition, residues 559–567 are involved in the crystallographic dimer interface (26), but the lack of differential uptake in our measurements suggests that they are either not involved in the solution-phase dimer interface or that the dynamics around these residues are too fast for our fastest deuterium uptake (12 s). Unfortunately, a gap in our HDX data in the region 63–70 precludes us from evaluating the assignment of these crystallographic interface contacts (26). Collectively, these biophysical studies suggest that rhβ-gal exhibits pH-dependent and concentration-dependent self-association and stability, with the enzyme being more prone to being an unstable monomer at low concentrations under neutral pH conditions and a stable dimer in an acidic environment.

### Gene therapy and ERT approaches can both augment β-gal activity and promote substrate clearance in patient cells for several weeks

**Figure 7.**
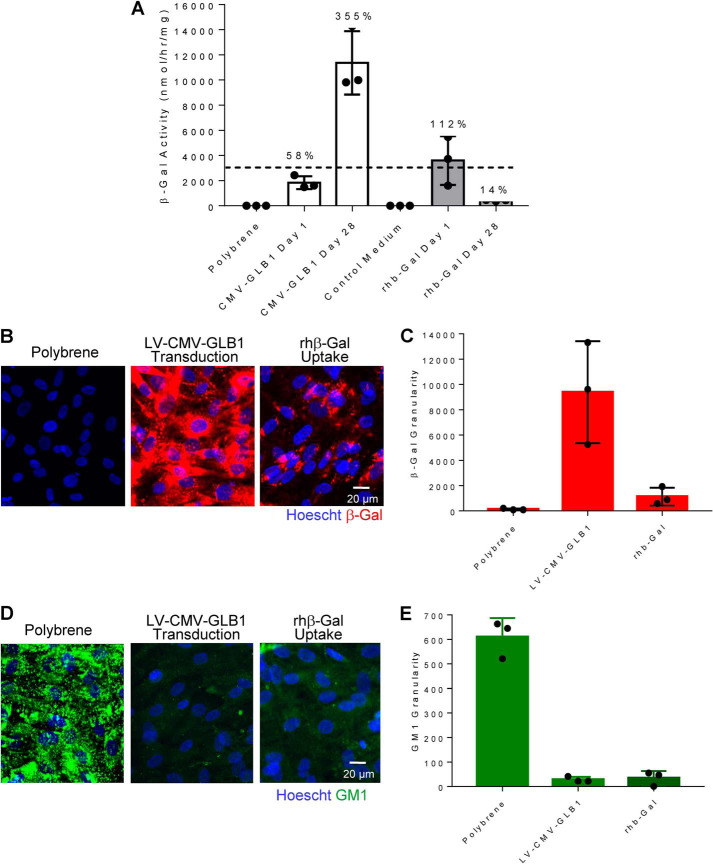
**Lentivirus-mediated GLB1 gene therapy and enzyme replacement therapy approaches can augment β-gal activity and promote substrate clearance for several weeks.** *A*, β-gal activity levels in GM1 gangliosidosis fibroblasts (GM05653) following transduction with LV-CMV-GLB1 (MOI = 5, *white bars*) for 24 h (day 1) or incubation with rhβ-gal for 24 h (100 nm, day 1 *gray bars*). Following each treatment, a separate group of cells were washed, incubated in growth medium, and then assayed for β-gal activity 28 days later. Enzyme activity detected in fibroblasts from a normal individual is indicated with a *black dashed line*. The level of enzyme activity present in patient cells after 1 or 28 days is indicated as a percentage of normal levels. *MOI*, multiplicity of infection. *B*, representative high-content images of β-gal immunofluorescence in control Polybrene-treated GM1 gangliosidosis patient fibroblasts (GM05653) or following transduction with LV-CMV-GLB1 (MOI = 5) or following cellular uptake of rhβ-gal (100 nm) at 28 days post-treatment. *C*, quantification of β-gal high-content imaging shown in *B. D*, representative high content images of GM1 ganglioside immunofluorescence in control untreated GM1 gangliosidosis fibroblasts (GM05653) or following transduction with LV-CMV-GLB1 (MOI = 5) or following cellular uptake of rhβ-gal (100 nm) at 28 days post-treatment. *E*, quantification of GM1 ganglioside high-content imaging shown in *D*. Three independent cultures in 96-well plates were imaged, with four images acquired per well at ×20 magnification. *Error bars* represent the standard deviation (S.D.).

Based on our biophysical studies, we predicted that chronic GLB1 overexpression may lead to instability of monomeric β-gal in the pH-neutral environment of the ER, where it could potentially trigger stress to this organelle. A lentivirus-mediated, CMV promoter–driven WT GLB1 gene therapy approach was used to chronically overexpress β-gal in GM1 gangliosidosis patient fibroblasts and compared with an intermittent ERT approach with a single 24-h exposure to rhβ-gal. β-Gal activity (Fig. 7*A*) and β-gal protein (Fig. 7*B*; see Fig. 7*C* for quantification) is not detected in primary fibroblasts from an infantile GM1 gangliosidosis patient. Consistent with the deficiency in β-gal, these cells accumulate β-gal substrates, as determined by CZE (Fig. 1*D*) and by high content imaging using a GM1 ganglioside polyclonal antibody (Fig. 7*D*; see Fig. 7*E* for quantification).

Chronic lentivirus-mediated WT GLB1 overexpression in GM1 gangliosidosis patient fibroblasts coincides with a time-dependent increase in β-gal activity (Fig. 7*A*) and β-gal protein levels as detected by immunofluorescence in patient cells over 28 days (Fig. 7*B*; see Fig. 7*C* for quantification), which coincides with almost complete GM1 ganglioside substrate clearance (Fig. 7*D*; see Fig. 7*E* for quantification), suggestive of successful delivery of functional β-gal to lysosomes. After 28 days of chronic GLB1 overexpression in GM1 gangliosidosis patient cells, the levels of β-gal activity reach supraphysiological levels corresponding to 355% of β-gal activity detected in fibroblasts from a normal individual (Fig. 7*A*). In comparison, cellular uptake of purified rhβ-gal for 24 h results in normalization of β-gal activity (Fig. 7*A*). Following withdrawal of enzyme from the uptake medium, lysosome-delivered β-gal activity levels slowly decay over a 28-day chase period (Fig. 7*A*), with the level of β-gal activity corresponding to ∼14% of normal β-gal activity (Fig. 7*A*), a level that is sufficient to maintain GM1 ganglioside substrate clearance over the entire 4-week duration of the experiment (Fig. 7*D*; see Fig. 7*E* for quantification). These results suggest that both gene therapy and ERT approaches can augment lysosomal β-gal activity sufficiently to promote substrate clearance for several weeks. Our results also suggest that β-gal levels do not need to reach supraphysiological levels to promote substrate clearance; only very small amounts of β-gal augmentation are sufficient to mediate near-to-complete GM1 ganglioside substrate clearance, with as little as 14% of normal residual β-gal activity (Fig. 7*A*) being sufficient to maintain substrate clearance in GM1 gangliosidosis patient cells for up to 4 weeks following cellular uptake of rhβ-gal (Fig. 7*D*; see Fig. 7*E* for quantification).

### Lentivirus-mediated GLB1 gene therapy in GM1 gangliosidosis patient fibroblasts activates an unfolded protein response, whereas ERT with purified rhβ-gal does not

**Figure 8.**
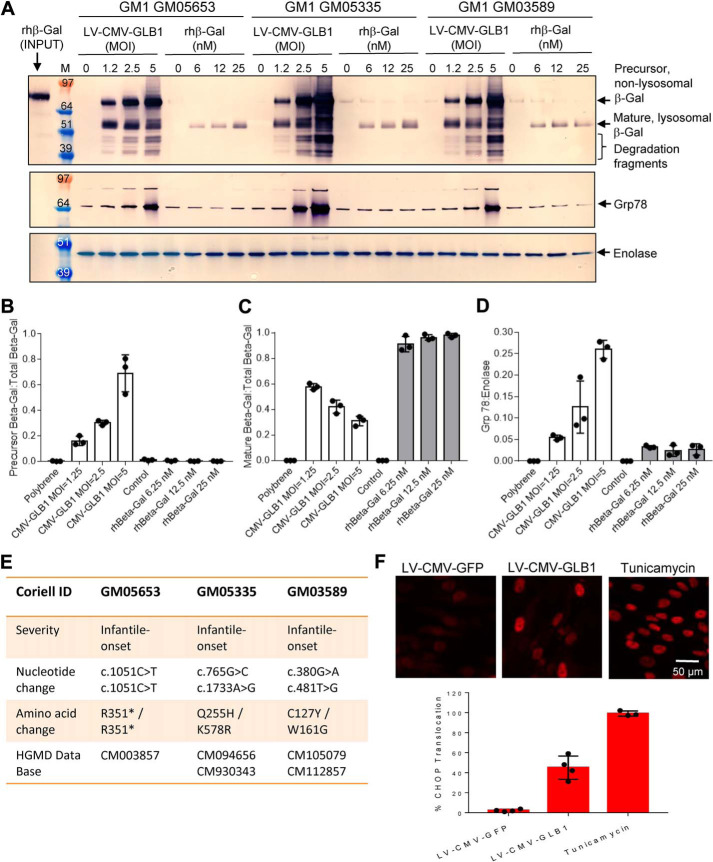
**Lentivirus-mediated GLB1 gene therapy in GM1 gangliosidosis patient fibroblasts activates the unfolded protein response, whereas ERT with purified rhβ-gal does not.** *A*, Western blots of cell lysates prepared from three infantile-onset GM1 gangliosidosis patient fibroblast lines 8 days after being transduced with LV-CMV-GLB1 (24-h transduction followed by 8-day chase; MOI = 1.25, 2.5, and 5) or 8 days after being incubated with purified rhβ-gal (24-h enzyme uptake followed by 8-day chase; enzyme concentration = 6.25, 12.5, or 25 nm). *B* and *C*, quantification of Western blots in *A*, with the amount of precursor β-gal (*B*) or mature lysosomal β-gal (*C*) expressed as a ratio of the total amount of precursor and mature enzyme detected in each treatment group for the three cell lines and expressed as individual values, *error bars* represent the standard deviation (S.D.). *D*, quantification of Grp78 protein levels detected in the three patient lines in *A*, standardized to the enolase loading control and expressed as individual values along with the S.D. *E*, genotype determination of the three GM1 gangliosidosis patient fibroblast lines described in *A*, with all mutations being described previously in the human gene mutation database (HGMD) and associated with infantile-onset GM1 gangliosidosis. *F*, representative high-content images of CHOP nuclear translocation observed in GM05653 cells transduced with LV-CMV-GFP (MOI = 5) or LV-CMV-GLB1 (MOI = 5) at 10 days post-transduction, with quantification of nuclear translocation for all treatments shown *below*. As a positive control for CHOP translocation, cells were treated with 2.5 μg/ml tunicamycin for 4 h. Three independent cultures in 96-well plates were imaged, with four images acquired per well at ×20 magnification.

Whereas both gene therapy and ERT approaches result in augmentation of β-gal activity and substrate clearance (Fig. 7), these results fail to address the subcellular distribution of β-gal. We therefore utilized Western blotting to distinguish between precursor, nonlysosomal β-gal and mature, lysosomal β-gal in GM1 gangliosidosis patient fibroblasts. Chronic lentivirus-mediated GLB1 overexpression in three infantile-onset GM1 gangliosidosis patient fibroblast lines (Fig. 8*A*; see Fig. 8*E* for patient genotypes) over a period of 8 days promotes a dose-dependent increase in the relative amounts of precursor nonlysosomal β-gal protein levels (Fig. 8*A*; see Fig. 8*B* for quantification), which coincides with a dose-dependent decrease in the relative amount of mature, lysosomal rhβ-gal protein levels (Fig. 8*A*; see Fig. 8*C* for quantification). These results are suggestive of retention of the overexpressed β-gal in a prelysosomal compartment and are in agreement with a previous report describing mislocalization of WT β-gal overexpressed in feline GM1 gangliosidosis fibroblasts, with the majority of enzyme co-localizing with the ER marker, PDI, suggestive of β-gal mislocalization and ER retention (18). Chronic GLB1 overexpression in all three GM1 gangliosidosis patient fibroblast lines also coincides with the appearance of smaller fragments on the β-gal Western blot (Fig. 8*A*), which were not characterized further. Based on our *in vitro* biophysical studies (Fig. 6), where we observed monomer-dimer equilibrium and instability at neutral pH (Fig. 6), we set out to determine whether accumulation of a prelysosomal pool of precursor β-gal in patient cells, presumably at neutral pH, activates an unfolded protein response, where ER-resident chaperones are up-regulated to maintain ER function during periods of cellular stress.

In agreement with the 2008 study by Martin *et al.* (18), we observed a direct correlation between chronic GLB1 transcriptional overexpression in GM1 gangliosidosis patient cells and activation of an unfolded protein response, with dose-dependent accumulation of precursor β-gal coinciding with increasing levels of precursor PDI protein being detected in all three GM1 gangliosidosis patient fibroblast lines after 4 days of chronic GLB1 overexpression (Fig. S4). Interestingly, after 8 days of chronic GLB1 overexpression, precursor PDI protein levels were no longer detected in the gene therapy–treated cells (Fig. S4), suggesting that up-regulation of precursor PDI levels is a transient response to GLB1 overexpression. Instead, chronic lentivirus-mediated GLB1 overexpression for 8 days in GM1 gangliosidosis patient fibroblasts coincides with dose-dependent increases in the levels of the ER-resident chaperone protein Grp78 (Fig. 8*A*; see Fig. 8*D* for quantification). The increased expression of Grp78 in GM1 gangliosidosis patient fibroblasts in response to virus-mediated GLB1 overexpression also appears to be time-dependent, with increased Grp78 protein levels only becoming apparent 7 days following transduction with LV-CMV-GLB1 (Fig. S5). Chronic lentivirus-mediated GLB1 overexpression for 8 days also coincides with nuclear translocation of C/EBP-homologous transcription factor (CHOP; Fig. 8*F*), an indication of irreversible damage caused by prolonged ER stress (23). Importantly, no up-regulation in Gpr75 protein levels (Fig. S5) or CHOP translocation (Fig. 8*F*) was detected in control GM1 gangliosidosis patient fibroblasts following lentivirus-mediated transduction with the GFP reporter gene (Fig. S5 and Fig. 8*F*). Collectively, our gene therapy studies in GM1 gangliosidosis patient cells are in agreement with Martin *et al.* (18), suggesting that GLB1 overexpression coincides with dose-dependent (Fig. 8 and Fig. S4) and time-dependent (Fig. S5) increases in early ER markers (PDI) and late ER markers (Grp78) of the unfolded protein response, as well as CHOP translocation, an indicator of prolonged ER stress.

In contrast to a chronic GLB1 gene therapy approach, an ERT approach with rhβ-gal cellular uptake for 24 h followed by an 8-day chase results in only the mature, lysosomal form of β-gal being detected in cell lysates by Western blotting (Fig. 8*A*; see Fig. 8*C* for quantification). Furthermore, an ERT approach with rhβ-gal does not activate an unfolded protein response at any of the doses tested, as determined by Western blotting for Grp78 protein levels (Fig. 8*A*; see Fig. 8*D* for quantification). Importantly, no evidence of an unfolded protein response was observed in ICV-ERT–treated mice, as determined by Western blotting with anti-Grp78 (Fig. 2*D*; quantified in Fig. 2*F*). Collectively, these results emphasize the potential for overexpressed β-gal to be retained in the ER of GM1 gangliosidosis patient cells following lentivirus-mediated GLB1 gene therapy, where accumulation of precursor, nonlysosomal rhβ-gal can activate the unfolded protein response and trigger ER stress. In contrast, cellular uptake of rhβ-gal by CI-MPR–mediated endocytosis from the cell surface results in direct delivery of the enzyme to acidified lysosomes, where it remains as a stable mature dimer.

## Discussion

**Figure 9.**
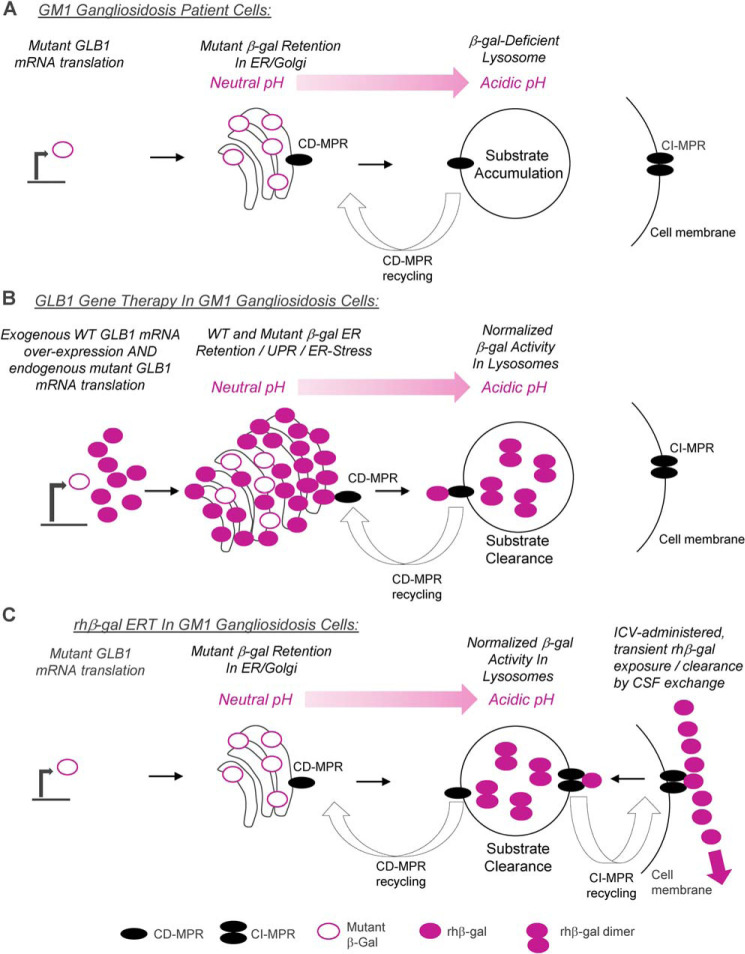
**Summary of the findings in this study.** *A*, endogenous mutant β-gal in GM1 gangliosidosis cells is prone to retention in the ER, which coincides with up-regulation of an unfolded protein response (18). *B*, GLB1 overexpression in feline GM1 gangliosidosis fibroblasts has previously been reported to promote mislocalization of the overexpressed WT β-gal, with the majority of enzyme being retained in the ER (18). In our hands, chronic GLB1 overexpression in GM1 patient cells coincides with partial retention of rhβ-gal in a prelysosomal compartment, presumably at neutral pH (Fig. 8). Our results, along with those of Martin *et al.* (18), suggest that accumulation of precursor, nonlysosomal rhβ-gal in the ER activates the unfolded protein response and triggers ER stress, suggestive of irreversible damage caused by a prolonged UPR. However, detection of the mature lysosomal form of rhβ-gal by Western blotting in gene therapy–treated patient cells (Fig. 8) coincides with substrate clearance for several weeks (Fig. 7), suggesting that a pool of overexpressed β-gal is reaching acidified lysosomes, where the enzyme likely resides as a stable dimer. In support of these findings, our *in vitro* biophysical studies suggest that rhβ-gal at low concentrations is predominantly a monomer and prone to destabilization at neutral pH, whereas at acidic pH, the enzyme is a stable dimer, independent of its concentration (Fig. 6). *C*, intermittent ERT with rhβ-gal results in direct and precise delivery of enzyme to acidified lysosomes, where the enzyme resides as a stable long-lived dimer, with a half-life of 9 days, which coincides with substrate clearance in patient cells (Figs. 1 and 7) and in the brain tissue of a mouse model of GM1 gangliosidosis (Fig. 3), without negatively impacting the ER (Figs. 2 and 8). CI-MPR–mediated endocytosis of rhβ-gal from the cell surface is dose-dependent and saturable, with very low-nanomolar doses of enzyme being sufficient to normalize β-gal levels (Fig. 1). Once the rhβ-gal cellular uptake capacity in lysosomes is reached in cells of the brain, the remaining extracellular ICV administered rhβ-gal is presumably cleared by CSF exchange, which occurs several times per day (27). In support of this, we observe normalization of β-gal activity levels in liver and bone marrow of ICV-ERT–treated KO mice (Fig. 5), suggestive of systemic exposure to the enzyme.

Our results suggest that the biophysical properties of β-gal, along with the therapeutic modality, should be considered when developing an effective treatment for GM1 gangliosidosis and that intermittent ICV-ERT dosing is a tunable therapeutic option that can safely and precisely deliver rhβ-gal to lysosomes to clear pathological lysosomal substrates and reverse neuropathology associated with the disease. Mechanistically, CI-MPR-targeted ERT efficiently targets rhβ-gal directly to acidified lysosomes of patient cells by cell surface receptor-mediated endocytosis. Following delivery to lysosomes of patient fibroblasts, rhβ-gal is converted to a mature, long-lived dimer that can normalize β-gal levels to promote substrate clearance for several weeks without negatively impacting the ER (Figs. 1 and 7; summarized in Fig. 9). Furthermore, as cell surface CI-MPR–mediated endocytosis and targeting of rhβ-gal to lysosomes of GM1 gangliosidosis cells approaches saturation (Fig. 1*B*), the excess enzyme presumably remains outside the cell, where it is cleared by cerebrospinal fluid exchange, which occurs several times per day (27). In support of this, we observed a perivascular pattern of β-gal activity *in situ* toward X-gal substrate immediately following ICV-ERT (Fig. 2*C*) along with normalization of β-gal activity in the liver (Fig. 5*A*) and bone marrow (Fig. 5*B*) of GLB1 KO mice following eight weekly ICV doses of rhβ-gal. These observations suggest that ICV administered enzyme that is not taken up by cells in the brain can reach systemic sites of pathology, with less life-threatening but severely debilitating skeletal pathology associated with GM1 gangliosidosis potentially being amenable to ICV-ERT dosing. Although no conclusion can be made after only eight weekly ICV doses of rhβ-gal in GLB1 KO mice, our results in a limited number of animals (Fig. 5*F*) warrant further studies to utilize urinary A2G2′ substrate levels as an indication of systemic exposure to rhβ-gal and a noninvasive biomarker of ICV-ERT efficacy.

Our biodistribution studies with rhβ-gal suggest that a single 100-μg dose of ICV administered rhβ-gal exhibits broad bilateral biodistribution throughout the brain of GM1 gangliosidosis mice as determined by utilizing an MS-based assay (Fig. 3*A*). Detection of β-gal activity *in situ* using X-gal substrate suggests that whereas the majority of lysosome-delivered enzyme activity is detected in perivascular regions at early time points (3 and 24 h post-ICV-ERT; Fig. 2*C*), lysosome-delivered β-gal activity in hippocampal neurons only becomes noticeable at the latest time point analyzed (48 h post-ICV-ERT; Fig. 2*C*), presumably by axonal transport. These observations help to explain how rhβ-gal biodistributes to lysosomes throughout the brain as well as the systemic circulation following ICV administration. Strikingly, weekly ICV-ERT dosing for 8 weeks is sufficient to promote near-to-complete substrate clearance in the brain (Fig. 3) and reversal of well-entrenched secondary neuropathology in GM1 gangliosidosis mice (Fig. 4). Although we did not perform reaccumulation ICV-ERT studies in GM1 gangliosidosis mice, our results in GM1 gangliosidosis patient fibroblasts suggest that a single low dose (3 nm) of rhβ-gal exhibits highly efficient CI-MPR–dependent cellular uptake and delivery to lysosomes, where the enzyme exhibits a long half-life (∼9 days), which coincides with substrate clearance for several weeks (Fig. 1, *G* and *H*) in the absence of an unfolded protein response (Fig. 8*D*). Furthermore, our results suggest that as little as 14% of normal residual β-gal activity remaining in GM1 gangliosidosis patient cells several weeks after ERT is sufficient to mediate complete clearance of lysosomal storage (Fig. 7*A*). These patient cell–based proof-of-concept results are in agreement with our previous findings in Sanfilippo B patient fibroblasts (9), suggesting that long-lived lysosomal enzymes do not need to reach supraphysiological levels in order to be therapeutic and that enzyme activity only needs to be augmented above a critical threshold of ∼10–30% of normal lysosomal enzyme activity levels to mediate substrate turnover. Future dose-ranging and reaccumulation studies in the GLB1 KO mouse model will help to titrate the ICV-ERT dose and dosing frequency for rhβ-gal in clinical trials for GM1 gangliosidosis patients.

Importantly, in terms of further development of ICV-ERT for GM1 gangliosidosis, we show here that purified rhβ-gal exhibits pH-dependent and concentration-dependent dynamic self-association, a property that has also been observed for insulin (28). We show that rhβ-gal can be formulated to high concentrations (20 mg/ml) in artificial CSF and at 1 mg/ml in a neutral pH test buffer (Fig. 6*H*), with loss of stability only being observed under neutral pH conditions over a period of several days at low concentrations (0.1 mg/ml) (Fig. 6*G*). In contrast, rhβ-gal appears to be a stable dimer under acidic pH conditions at low concentrations (0.1 mg/ml) (Fig. 6*G*). These biophysical properties of rhβ-gal become important following ICV administration, when the enzyme becomes rapidly diluted as it diffuses further from the injection site in extracellular fluids, which are presumably at neutral pH. Following ICV-ERT, we observed mature, lysosome-delivered β-gal protein by Western blotting in brain, liver, and bone marrow of treated GLB1 KO mice, suggestive of the enzyme being successfully delivered to the acidified lumen of lysosomes at several sites of pathology (Figs. 3*C* and 5 (*A* and *B*)). β-Gal activity is also detected *in situ* in neurons of GLB1 KO mice following a single ICV dose of rhβ-gal, further suggestive of successful delivery to acidified lysosomes (Fig. 2*C*). In our patient cell–based ERT studies, we show that rhβ-gal is targeted directly to acidified LysoTracker Red^+^ lysosomes by CI-MPR–mediated endocytosis from the cell surface (Fig. 1*C*), where the enzyme decays very slowly over a period of 6 weeks (half-life = 9 days; Fig. 1*F*). These results collectively suggest that an ERT approach can therefore exploit the biophysical properties of β-gal to directly target rhβ-gal to acidified lysosomes, where the enzyme can exist at low concentrations as a long-lived dimer. At the same time, from a formulation standpoint, our biophysical studies suggest that rhβ-gal can be formulated to high concentrations in a neutral pH buffer such as artificial CSF without any noticeable loss in stability.

Our results suggest that whereas ganglioside and oligosaccharide substrates that accumulate in the absence of β-gal contribute to fatal and devastating neurological disease progression in GM1 gangliosidosis, gene therapy approaches to add back the missing GLB1 gene product also have the potential to promote toxicity. We utilized Western blotting to demonstrate that chronic lentivirus-mediated, CMV promoter–driven GLB1 overexpression in GM1 gangliosidosis patient fibroblasts leads to accumulation of the precursor form of rhβ-gal in a prelysosomal compartment, where it activates an unfolded protein response and ER stress (Fig. 8). Gene therapy experiments utilizing the GFP reporter gene exclude the possibility of the ER-associated toxicity arising from the lentivirus vector but rather the GLB1 gene product itself (Fig. 8*F* and Fig. S5). Whereas the mechanism by which GLB1 overexpression activates an unfolded protein response and promotes ER stress in patient cells was not investigated in this study, our biophysical studies with rhβ-gal suggest that this glycosidase exhibits dynamic self-association under neutral pH conditions and is more prone to instability, particularly at lower concentrations (Fig. 6). This would suggest that retention of overexpressed β-gal in the pH neutral compartment of the ER could potentially promote instability of the enzyme, which in turn may activate the unfolded protein response and trigger ER stress (Fig. 8; summarized in Fig. 9).

These gene therapy results in patient cells emphasize the importance of carefully monitoring the ER system with straightforward methods when developing gene therapies and, in the case of GM1 gangliosidosis, titrating the extent and duration of GLB1 dosage to avoid accumulation of transcriptionally up-regulated β-gal in the ER. Our GLB1 gene dosage studies in Fig. 8*A* suggest that gene therapy can be a safe and effective treatment for GM1 gangliosidosis, provided that the strength of the promoter driving GLB1 transcription and virus dose is carefully titrated to prevent its overexpression. In support of this, Martin *et al.* (18) demonstrate correct rhβ-gal localization to lysosomes in feline GM1 gangliosidosis fibroblasts expressing physiological levels of rhβ-gal and only observe mislocalization and ER retention of rhβ-gal in cells expressing above normal levels of the enzyme. In our hands, we observed a dose-dependent (Fig. 8) and time-dependent (Fig. S5) up-regulation of an unfolded protein response in patient cells overexpressing β-gal, suggesting that careful tuning of GLB1 expression levels will help to avoid ER stress and can lead to a potentially safe and effective gene therapy for GM1 gangliosidosis.

An additional safety issue that remains to be investigated for the management of GM1 gangliosidosis patients undergoing β-gal augmentation therapy is the potential for continual β-gal delivery to lysosomes to disrupt a multienzyme complex that exists between protective protein cathepsin A (PPCA), neuraminidase 1 (Neu1), and β-gal (29). Whereas Neu1 is strictly dependent upon the chaperone function of PPCA for its stability in lysosomes, β-gal is only partially dependent (29–31). Our companion manuscript, also published in this issue of JBC, demonstrates that lysosome-targeted b-galactosidase negatively regulates neuraminidase 1 (NEU1) and promotes NEU1 deficiency in GM1 gangliosidosis (40). Chronic cellular uptake of purified recombinant human b-Gal (rhb-Gal) or chronic lentiviral-mediated GLB1 overexpression in GM1 gangliosidosis patient fibroblasts coincides with profound secondary NEU1 deficiency. In contrast, a regimen of intermittent enzyme replacement therapy dosing with rhb-Gal, followed by enzyme withdrawal, is sufficient to augment b-Gal activity levels in GM1 gangliosidosis patient fibroblasts and in a mouse model of GM1 gangliosidosis without promoting NEU1 deficiency (40). This is particularly important, given that Neu1 deficiency is associated with increased lysosomal exocytosis (32) and onset of the neurodegenerative lysosomal storage diseases sialidosis and galactosialidosis (29).

In summary, our results suggest that the biophysical properties of β-gal should be considered when developing a safe and effective treatment for GM1 gangliosidosis. An ERT approach in patient cells and a mouse model of the disease circumvents the pH-neutral environment of the ER and directly delivers β-gal to acidified lysosomes, where the enzyme resides as a stable dimer that can clear pathological substrates (Fig. 9*C*). The long half-life of lysosome-delivered rhβ-gal (9 days) may permit less frequent ICV dosing, which would need to be tested in future preclinical dose-ranging and reaccumulation studies. In contrast to the ERT paradigm, a gene therapy approach to transcriptionally up-regulate GLB1 expression and augment β-gal levels in patient cells highlights the potential for overexpressed β-gal to mislocalize and be retained in the ER, presumably at neutral pH, where the enzyme promotes an unfolded protein response and triggers ER stress (Fig. 9*B*). This is likely to be due to dynamic self-association and reduced stability of β-gal under neutral pH conditions.

## Experimental procedures

### Production of rhβ-gal in CHO cells

Research-grade recombinant human β-gal protein was purified from supernatants of stably transfected CHO cells generated with the GS Mammalian Gene Expression System (Lonza Biologics). Briefly, the CHO GSKO cells, grown in suspension in CD CHO medium (Thermo Fisher Scientific), were transfected by electroporation with the pXC 17.4/β-gal expression plasmid. The transfected cells were directly plated in 96-well plates in the absence of glutamine. The resulting colonies were screened by the β-gal enzyme activity assay (see below), and the clones expressing the highest amount of the protein were isolated and expanded.

The rhβ-gal protein was produced in shaker flasks or 10-liter working volume bioreactors (Applikon Biotechnology). For fed-batch productions, the rhβ-gal–expressing CHO research clone was seeded at 0.5 × 10^6^ cells/ml in CD CHO medium or blended media containing Efficient Feed B (Thermo Fisher Scientific). The pH set point of 6.9 was controlled by CO_2_ and 0.5 m NaHCO_3_ control loops. The dissolved oxygen set point of 30% was controlled by cascade of clean dry air and O_2_ mass flow controllers. Additional feeds and glucose were provided as glucose levels fell below 2 g/liter. Cell culture fluid was harvested on day 14 postinoculation, filtered, and stored at −80 °C until the purification process.

### Purification of rhβ-gal

Recombinant human β-gal protein was purified via anion-exchange, hydrophobic interaction chromatography and multimodal chromatography. Diluted harvest culture fluid was applied to a GigaCap Q 650M (Tosoh Bioscience) column in 50 mm Tris, pH 7.5, and was eluted from the column in a linear gradient of NaCl. The GigaCap Q eluate was then loaded onto a butyl-Sepharose 4 Fast Flow (GE Healthcare) column in 1 m (NH_4_)_2_SO_4_, followed by an elution step with decreasing (NH_4_)_2_SO_4_ concentration. The butyl eluate was loaded onto a Capto Adhere column (GE Healthcare) in flow-through mode with 50 mm acetate/phosphate, 135 mm (NH_4_)_2_SO_4_, pH 7. During the purification process, samples were analyzed by Western blotting and β-gal activity determination using 4-methylumbelliferone (4MU) substrate, reversed-phase and size-exclusion HPLC, and multiangle light scattering. Two small-scale production and purification trains were initially performed (lots 1 and 2), with a large-scale campaign used to generate ∼300 mg of rhβ-gal material (lot 3), which was used for all experiments described in this work. The final recovery of β-gal was 18.3%, with a yield of 316 mg, as determined by absorbance at 280 nm. The specific activity of purified rhβ-gal was determined to be 2.6 units/mg, with an endotoxin level of 0.02 endotoxin units/mg. Purified rhβ-gal was diafiltered and concentrated to 20 mg/ml in a solution of artificial CSF (aCSF; 1 mm Na_2_PO_4_/NaH_2_PO_4_, 148 mm NaCl, 3 mm KCl, pH 7.1) and stored as 1-ml aliquots at −80 °C.

### Biophysical characterization of purified β-gal

SV-AUC and SE-AUC were performed with a ProteomeLab XL-I (Beckman-Coulter) equipped with absorbance optics and an An-50 Ti eight-hole rotor. SV-AUC experiments were performed at 48,000 rpm and monitored at 230 or 280 nm. Data were analyzed with the *c*(*s*) method as implemented in the program SEDFIT (33). SE-AUC experiments were carried out at 12,000 and 14,000 rpm and monitored at 230, 280, and 300 nm. Equilibrium at both rotor speeds was established by evaluating the RMS difference between scans. Data were individually and globally analyzed with the following models as implemented in the program SEDPHAT: single noninteracting species and monomer-dimer equilibrium (34). DSF was performed by monitoring the change in intrinsic fluorescence as a function of temperature with the Uncle platform (Unchained Labs). Thermograms were analyzed in the Uncle analysis software package. HDX was performed with the HDX-2 system for sample handling and chromatography and the Synapt G2Si tandem mass spectrometer for data acquisition (both from Waters Corp., Milford, MA). Exchange-in was performed in triplicate at 12 and 120 s. Data analysis was performed in DynamX (version 3.0.0, Waters) and results were illustrated in PyMOL (version 1.4.8.1, Schroedinger, LLC) For these biophysical experiments, the two examined conditions contained the following components: 20 mm sodium phosphate (pH 7.4) or 20 mm sodium acetate (pH 5), 150 mm NaCl, and 3 mm KCl. For all experiments, β-gal was extensively dialyzed overnight to help ensure complete buffer exchange.

### GM1 gangliosidosis patient fibroblast lines

Infantile-onset GM1 gangliosidosis patient fibroblasts, GM05653 (35), GM03589 (36), and GM05335 fibroblasts were obtained from the Coriell Institute for Medical Research and used as a cell-based model of GM1 gangliosidosis. Fibroblasts from a normal individual, GM008339, were also obtained from Coriell. Cells were grown in GM1 complete medium Eagle's minimum essential medium (ATCC, catalog no. 30-2003) supplemented with 15% fetal bovine serum (not heat-inactivated), 1× penicillin/streptomycin (Thermo, catalog no. 15140122), and 1× Glutamax (Thermo, catalog no. 35050-061). Whereas β-gal activity was not detected in these patient lines, the GM05653 and GM03589 lines stored greater amounts of GM1 ganglioside and glycan substrates by capillary zone electrophoresis, when compared with the GM05335 cell line. Genotyping of the GLB1 coding region in each cell line was performed by Greenwood Genetic Center (Greenwood, SC).

### Analysis of rhβ-gal uptake in GM1 gangliosidosis patient fibroblasts

For cellular uptake experiments, GM1 GM05653 fibroblast cells were seeded at 12,500 cells/well into 96-well plates. Approximately 24 h after seeding, GM05653 cells were incubated with increasing concentrations (dose range of 0.05 → 50 nm) of rhβ-gal with or without 8 mm mannose 6-phosphate disodium salt hydrate (Sigma, catalog no. M6876). In pulse-chase experiments, cells were allowed to uptake enzyme for ∼24 h, after which medium containing enzyme was removed, cells were washed, and fresh complete medium was added to chase lysosomal rhβ-gal.

Cell lysates were prepared in mammalian protein extraction reagent (MPER; Thermo, catalog no. 78501). Uptake of rhβ-gal into GM05653 fibroblasts was monitored by Western blotting cell lysates with β-galactosidase antibody from Novus (catalog no. NBP2-45731). β-Gal uptake was also monitored by activity against 4-methylumbelliferyl-β-d-galactopyranoside fluorogenic substrate (4MU-GAL; Sigma, catalog no. M1633). Cell lysates or input media containing β-gal were incubated with 4 mm 4MU-GAL in assay buffer (100 mm sodium citrate, 250 mm NaCl, 1% Triton X-100, 0.2% BSA, pH 4.5) for 40 min at 37 °C. The reaction was stopped with stop buffer (0.5 m glycine, 0.3 m NaOH, pH 10.3), and fluorescence was read on a Spectramax i3 plate reader (Molecular Devices) with excitation emission of 355/460 nm, respectively. Serial dilutions of 4MU (Sigma, catalog no. 1381) were used to establish a standard curve and calculate β-gal activity in samples. To determine the *K*_uptake_, enzyme activity was plotted using Michaelis–Menten analysis within GraphPad Prism software. For half-life determination, GM05653 cells were incubated with a low-nanomolar dose of rhβ-gal (3.125 nm) for 18 h and then washed several times to remove any noninternalized enzyme, and fresh growth medium was added to chase. β-Gal activity was assayed roughly every 1–2 weeks, over a period of 6 weeks. Half-life was determined by plotting β-gal activity into GraphPad Prism software and using nonlinear exponential decay regression.

Alternatively, β-gal uptake was monitored by immunofluorescent staining and microscopy. β-Gal was directly conjugated with Alexa Fluor 488 (AF488) prior to uptake. For the AF488 conjugation, β-gal was reacted with a 5-fold molar excess of Alexa Fluor 488 5-SDP Ester (Thermo, catalog no. A30052) and labeled according to the manufacturer's protocol, yielding ∼1.2 fluorophores/molecule. AF488 conjugation did not affect rhB-gal activity.

A GLB1 mAb was also generated in-house using rhβ-gal (lot 3), which was used for high-content imaging. Granularity was calculated as total granule area in each acquired image, multiplied by the average granule intensity, divided by the total number of cells imaged. A minimum of three independent cultures in 96-well plates were imaged, with four images per well acquired at ×20 magnification. GM05653 fibroblasts were incubated with 25 nm rhβ-gal-AF488 for 24 h. Cells were then rinsed and incubated with 0.5 μm LysoTracker Red (Thermo, catalog no. L7528) prior to fixation to stain acidified lysosomes. Cells were imaged on an ImageXpress Micro XLS high-content microscope (Molecular Devices) at ×40 objective obtaining four sites per well. β-Gal activity was also detected *in situ* following cellular uptake of rhβ-gal using X-gal substrate, according to the manufacturer's kit protocol (Cell Signaling Technology, catalog no. 9860) with the X-gal staining solution being adjusted to pH 4.5 for detection of β-gal specifically in lysosomes. Stained cells were imaged on the ImageXpress microscope at ×40 objective using transmitted light.

### Substrate detection in patient cells by GM1 immunostaining and high-content imaging

To test substrate accumulation in GM05653 patient cells, we used a commercially available GM1 antibody for immunostaining. Briefly, GM05653 cells were grown for 7–10 days to permit substrate accumulation and then fixed (4% paraformaldehyde in PBS) and permeabilized using normal goat serum (Thermo, catalog no. 50062Z) with 0.01% Triton X-100. Cells were then blocked in normal goat serum and then stained with an anti-GM1 antibody (Abcam, catalog no. ab23943, diluted 1:250 in normal goat serum) overnight at 4 °C. Cells were imaged on an ImageXpress high-content microscope as described previously. Granular immunostaining with anti-GM1 antibody was quantified using a granularity algorithm within MetaMorph software. GM1 granularity was calculated as total granule area multiplied by average granule intensity divided by the number of cells and expressed as arbitrary units.

### Substrate detection in GM1 gangliosidosis patient cells by glycan profiling

Cell extracts from GM1 gangliosidosis human patient fibroblast cell lines were found to accumulate two distinct classes of substrates in lysosomes after extended culture times due to the absence of lysosomal β-gal activity: GM1 gangliosides and oligosaccharides (glycans) arising from cellular glycoprotein turnover. Profiling glycans derived from GM1 gangliosides require releasing oligosaccharides enzymatically from the lipid backbone using the endoglycosidase ceramide glycanase, followed by a precapillary derivatization with a charged fluorophore. This labeling reaction occurs through reductive amination, a reaction of the amino group of the fluorophore dye and aldehyde of the reducing termini generated on the released GM1 glycan. In this protocol, other oligosaccharides with reducing termini resulting from the incomplete digest of cellular glycoproteins in the lysosome were also labeled. Once labeled, the oligosaccharides were separated by CZE and detected by laser-induced fluorescence.

Alternatively, assaying only a subset of oligosaccharide structures is done by directly labeling the β-gal–deficient cell extracts and omitting the ceramide glycanase endoglycosidase treatment (*i.e.* assay of glycans that resulted from incomplete digest of cellular glycoproteins). Glycan structures were determined in three ways: 1) by direct comparison with HILIC-MS–identified oligosaccharides and migration position by CE, 2) by exo-glycosidase digestion of peaks to observe expected shifts in migration times, and 3) by co-migrating glycan standards.

Cell extracts were prepared as follows. Internal CZE standard (3′-sialyl-*N*-acetyllactosamine, Glyco AD-01014; molecular weight 656.2) was reconstituted to 1 mg/ml in sterile water to obtain a 1.5 mm solution. 50-μl aliquots were prepared and stored at −20 °C. In preparation of samples for CZE, 3′-syalillactosamine standard was diluted 1:100 in mammalian protein extraction reagent solution. Cells were lysed with 3′-syalillactosamine mammalian protein extraction reagent solution. 25 μl of each cell extract was digested with 3 μl of ceramide glycanase (QA-bio, catalog no. LZ-CER-HM) overnight at 37 °C. The reactions were then dried in a SpeedVac. The recovered oligosaccharides were derivatized by reductive amination with 2 μl of 50 mm 8-aminopyrene-1,3,6-trisulfonic acid (APTS, Sciex, catalog no. 501309) and 7 μl of 1 m sodium cyanoborohydride (Sigma, catalog no. 296813), followed by a vigorous mixing and incubation at 37 °C overnight. The following day, the oligosaccharides were resuspended in 10 μl of water followed by another vigorous mixing. Excess APTS dye was removed from the labeling mixture by packing a low-speed Sephadex G10 gel column (GE Healthcare, catalog no. 17-0010-01) followed by the addition of the glycan-labeling reaction and then spun for 3 min at 1000 rpm in a bucket centrifuge and recovery of glycans in the eluate. CZE was performed on the CESI 8000 Plus (Sciex) using laser-induced fluorescence. The laser excitation wavelength was 488 nm. A 65-cm N-CAP–coated capillary with a 50-μm inner diameter was used along with a Sciex kit-supplied N-CAP buffer (Sciex, catalog no. 477600). The capillary was pre-rinsed with buffer at 40 p.s.i. for 1 min. A “water pillow” was injected at 0.5 p.s.i. for 20 s, followed by sample injections at 0.8 p.s.i. for 5.0 s. A second water pillow was injected at 0.1 p.s.i. for 10 s following the sample loading. Separation voltage was set to 21 kV for 20 min. The capillary temperature was set to 25 °C. The raw data were processed using 32 Karat software.

### Evaluation of the ER system following β-gal augmentation in patient fibroblasts

LV-CMV-GLB1 and LV-CMV-GFP plasmids were designed, constructed, and used for large-scale production and ultrapurification of lentivirus particles by VectorBuilder (Santa Clara, CA). Virus was stored in 100-μl aliquots at −80 °C, with a titer of 10^9^ transduction units/ml, as determined by VectorBuilder. Patient fibroblasts were plated into 96-well plates at 12,500 cells/well and transduced with lentivirus in the presence of 0.5 μg/ml Polybrene at varying multiplicity of infection (MOI). Alternatively, patient cells were incubated with rhβ-gal at varying concentrations. Following 24 h of virus transduction or cellular uptake with enzyme, cells were washed several times and then assayed for β-gal activity (day 1). Alternatively, cells were chased in growth medium for up to 28 days, with β-gal activity assayed in duplicate plates at day 7 and day 28.

At day 8, cells were lysed using mammalian protein extraction reagent and analyzed by Western blotting for β-gal protein levels using β-galactosidase antibody (Novus, catalog no. NBP2–45731) or Grp78 BiP antibody (Abcam, catalog no. MA1-250).

CHOP distribution was analyzed at day 10 by immunofluorescence using a CHOP mAb (9C8; Thermo Fisher Scientific). Stained cells were analyzed by high-content imaging using the ImageXpress. In each well, four individual images were acquired at ×40 magnification. Images were acquired in the red (CHOP) and blue (nuclei) channels, and the number of nuclei was recorded as an indicator of cell viability. The nuclei in the blue channel were masked and used for analyzing the extent of CHOP translocation from the cytosol to the nucleus in the red channel. The nuclei that co-localized with endogenous CHOP were scored as translocation events and expressed as a percentage of the total number of cells imaged in each field. Tunicamycin (2.5 μg/ml), a known inducer of CHOP translocation (22), was used as a positive control for ER stress.

### Animals and ICV cannula implantation

The GLB1 null mouse (GLB1 KO) was generated as described by Hahn *et al.* (21). Founder mice were transferred from St. Jude Children's Research Hospital (Memphis, TN) to the Jackson Laboratories (Bar Harbor, ME) for re-derivation prior to importation to the BioMarin vivarium at the Buck Institute for Research and Aging (Novato, CA). Mice for this study were bred in-house from these re-derived animals. GLB1 KO mice were generated by mating heterozygous female (GLB1^+/−^) mice with male GLB1^−/−^ mice. C57BL/6J mice were bred for use as WT controls. The studies conducted herein were approved by the Institutional Animal Care and Use Committee of the Buck Institute. Briefly, a permanent cannula was surgically placed into the left lateral ventricle of the brain of each mouse 5 days prior to the initiation of each study. ICV infusions (5-μl total volume over a period of 15 min) were administered via the implanted cannula to deliver either rhβ-gal (100 μg) or vehicle (aCSF; 1 mm Na_2_PO_4_/NaH_2_PO_4_, 148 mm NaCl, 3 mm KCl, pH 7.1). For biodistribution and efficacy studies, brains were bisected along the superior sagittal sinus. The left hemisphere (ipsilateral to the infusion site) was processed for biochemical analysis. The right hemisphere (contralateral to the infusion site) was fixed in 10% paraformaldehyde for 24 h prior to processing for formalin-fixed, paraffin-embedded tissue sectioning. Additional brains were also frozen for a histology-based frozen tissue β-gal activity assay.

### Animal experimental design

To evaluate how early GLB1 KO mice bred at BioMarin begin to indicate detectable levels of neuropathology, brains from untreated WT or GM1 mice at 8 weeks of age were collected for subsequent analysis (*n* = 3–4). This is the earliest time that ICV infusions are approved for use in mice. To ensure the detection and localization of β-gal in treated mice, brains from 16-week-old WT and GM1 mice given a single, unilateral infusion of β-gal or vehicle (*n* = 3) were collected at 3 h post-treatment for MS analysis of β-gal protein levels. Additional brains were harvested at 3, 24, and 48 h to evaluate β-gal activity *in situ* using X-gal substrate (see below). To evaluate efficacy of ICV-ERT, two study designs were implemented. First, GM1 mice were treated twice a week starting at 8 weeks of age for 2 weeks with a single 100-μg infusion of β-gal until 10 weeks of age (2-week PoC; four doses total; shorter, more frequent exposure to enzyme) with the left and right hemispheres being used for β-gal activity, ganglioside, and glycan measurements. Second, GM1 mice were treated once a week starting at 12 weeks of age with a single infusion of β-gal until 20 weeks of age (8-week PoC; eight doses total; longer, less frequent exposure to enzyme), with only the left hemisphere being used for β-gal, ganglioside, and glycan measurements and the right hemisphere being fixed and used for histology (see below). Brains were collected 24 h after the last infusion and processed for downstream analysis.

### Immunohistochemistry and immunofluorescence staining of brain tissue

Brain tissues were immersion-fixed in formalin and embedded in paraffin. For the majority of the IHC analyses, 7-μm-thick sagittal sections were taken at approximately the superior sagittal sinus (sagittal midline) delineating the left and right brain hemispheres. Region matching for the sagittal sections was conducted utilizing the lateral ventricle, hippocampus, thalamus, pons (pontine reticular nucleus), and the VIa lobule of the central cerebellar region, corresponding to position 164 of the mouse Allen Brain Atlas. For the biodistribution analysis, additional studies utilizing 7-μm coronal sections were taken at approximately the injection site (left hemisphere lateral ventricle). Region matching for the coronal sections was conducted using the lateral ventricles as a reference point, corresponding to position 218 of the mouse Allen Brain Atlas. Sections were immunostained with antibodies against LAMP2 (Abcam, 25339, 1:500), GFAP (Sigma, g9269, 1:4000) or IBA1 (Millipore, MABN92, 1:250). For antigen retrieval, slides were immersed in Discovery CC1 solution (Ventana, catalog no. 950-500) for 30 min at 95 °C. The blocking buffer consisted of a 2% NDS, 0.1% BSA, and 0.3% Triton solution in 1× TBS. Donkey anti-rabbit IgG (H+L) highly cross-adsorbed secondary antibody conjugated to Alexa Fluor 488 (Thermo Fisher Scientific, A-21206, 1-250) was used to detect anti-GFAP antibody. Anti-LAMP2 antibody was detected by a Donkey anti-mouse IgG (H+L) highly cross-adsorbed secondary antibody conjugated to Alexa Fluor 555 (Thermo Fisher Scientific, A-31570, 1-500). Tris-buffered saline was used for all washes. Chromogenic staining of LAMP2 was performed due to observed high nonspecific signal when utilizing fluorescence-based immunohistochemistry. The principal methodology was comparable with fluorescence IHC. In addition, peroxide block (Bloxall, Vector Laboratories) was utilized to reduce endogenous peroxidase activity. The IMPRESS Rat polymer detection kit for chromogenic IHC was utilized to resolve the 3,3′-diaminobenzidine–labeled LAMP2 signal (Vector Laboratories). After staining, whole sections were scanned on a Zeiss Axio Scan.Z1 using a ×20 Plan-Apo objective (Zeiss). Regions of interest include the cerebral cortex (somatomotor areas 2, 3, 5, and 6A), hippocampus, thalamus, and the pons (pontine reticular nucleus). Representative confocal images were acquired on a Leica TCS SP8 confocal microscope with an HC PL APO ×40/1.30 or ×63/1.4 oil objective and 1-airy unit pinhole diameter (Leica Microsystems).

### Measurement of β-gal activity in situ in frozen tissue sections

To evaluate region-specific development of β-gal detection after a single infusion, the ipsilateral hemispheres of sagittal bisected brains were collected 24 h after infusion, frozen at −80 °C, and embedded in OCT for frozen sectioning. Brains were sectioned at 15 μm onto Superfrost Plus slides (Thermo Fisher Scientific) and fixed in 0.25% glutaraldehyde in PBS for 15 min. The slides were then rinsed in PBS and utilized in an X-gal–based substrate staining kit (Senescence β-galactosidase staining kit, Cell Signaling, catalog no. 9860). The manufacturer's suggested protocol was utilized with slight modifications. The pH of the staining solution was adjusted to 4.5 with the volumes proportionally normalized for the size of mouse sagittal brain sections. After signal development, whole sections were scanned as before, with similar regions of interest extracted for sagittal sections. Signal isolation and analysis was conducted via Photoshop (Adobe) and ImageJ. For both fluorescence and chromogenic-based IHC, the percentage of area of the total adjusted signal was utilized for downstream analysis. All data were analyzed via GraphPad Prism. Analysis of variance with Tukey post hoc testing was utilized to analyze the variance between each of the treatment groups. *p* < 0.05 was determined to be statistically significant.

### Detection and quantification of human β-gal in mouse brain homogenate using LC-parallel reaction monitoring (PRM)–based targeted MS

All solvents were HPLC-grade from Sigma-Aldrich, and all chemicals where not stated otherwise were obtained from Sigma-Aldrich. Mouse left and right brain hemispheres were each dissected into the following regions: olfactory bulb (OFB), cerebral cortex (CBX), hypothalamus and septum (HS), cerebellum (CB) and mid-brain (MB, containing pons and medulla) and frozen individually. Frozen tissue samples were transferred to homogenization tubes preloaded with zirconium oxide beads (REDE-RNA, Next Advance), and a different volume of cold HPLC-grade water (Sigma-Aldrich) was added to each sample (150 μl for CB, 90 μl for OFB, 360 μl for HS, 240 μl for MB, 480 μl for CBX). Samples were homogenized with a Bullet Blender (Next Advance) in a cold room (4 °C). Subsequently, 4 parts of Biognosys lysis buffer (Zurich, Switzerland) were added to 1 part of brain water homogenate. The protein concentration of all samples was determined using a BCA assay (Thermo Fisher Scientific). The samples were then shipped to Biognosys (Zurich, Switzerland) for LC-PRM MS analysis. Samples were reduced using Biognosys' Reduction and Alkylation solution and digested overnight with sequencing grade modified trypsin (Promega) at a protein/protease ratio of 50:1. The digested peptides were cleaned up for MS using C18 MacroSpin columns (SMM SS18V, TheNestGroup) according to the manufacturer's instructions. The cleaned up peptides were dried down using a SpeedVac system and redissolved in LC solvent A (1% acetonitrile in water with 0.1% formic acid (FA)) containing iRT-peptide mix (Biognosys) for retention time calibration. Peptide concentration was measured at 280 nm with a SPECTROstar® Nano spectrophotometer (BMG Labtech). Custom stable isotope-labeled reference peptides (Maxi SpikeTides^TM^_QL_AAA, ±10% quantification precision, >95% purity) were obtained from JPT (Berlin, Germany): AYVAVDGIPQGVLER, TEAVASSLYDILAR, and TVGAALDILCPSGPIK. For the absolute quantification of human β-gal, the equimolar pool of the three peptides was used and added to the final peptide samples at known concentration as internal standards.

For the quantification of human β-gal peptides by LC-PRM (1 μg/sample), samples were injected to an in-house packed C18 column (ReproSil-Pur120C18AQ, 1.9 μm, 120-Å pore size; 75-μm inner diameter, 50-cm length, New Objective) on a Thermo Scientific Easy nLC 1200 nano-LC system. LC solvents were as follows: A, 1% acetonitrile in water with 0.1% FA; B, 15% water in acetonitrile with 0.1% FA. The LC gradient was 5–40% solvent B in 50 min followed by 40–90% B in 2 min and 90% B for 12 min (total gradient length was 64 min). LC-PRM runs for peptide quantification were carried out on a Thermo Scientific Q Exactive mass spectrometer equipped with a standard nano-electrospray source. Collision energies were 25 eV according to the vendor's specifications.

An unscheduled run in PRM mode was performed before data acquisition for retention time calibration using Biognosys' iRT concept as described (37). The acquisition window was 4 min. Signal processing and data analysis were carried out using SpectroDive^TM^ 7.0 (Biognosys), based on mProphet (38). A *Q*-value filter of 1% was applied. The absolute quantification was determined by comparing the abundance of the known internal standard peptides with the endogenous peptides. The ratio of the areas under the curve (between the endogenous and reference peptide) was used to determine the absolute levels of human β-gal in the samples. Results were recalculated for fmol of human β-gal (using the molecular mass of 76,000 Da from the UniProt database) per mg of tissue.

### Assays of β-gal enzyme activity in tissues

Frozen tissue samples (∼200 mg of liver tissue or brain hemispheres weighing 150–300 mg each) were transferred to homogenization tubes preloaded with zirconium oxide beads (REDE-RNA, Next Advance), and 600 μl of cold HPLC-grade water (Sigma-Aldrich) was added to each sample. Samples were homogenized with a Bullet Blender (Next Advance) in a cold room set to 4 °C. An aliquot (300 μl) of this homogenate was removed for ganglioside and glycan analysis and neuraminidase activity measurements. The remaining tissue homogenate in the lysing tubes was homogenized again after the addition of 420 μl of chilled T-Per buffer (Thermo Fisher Scientific) with protease inhibitor mixture (Thermo Fisher Scientific). This homogenate was transferred to a new tube containing an additional 300 μl of T-Per with protease inhibitors. The T-Per homogenate samples were centrifuged in a refrigerated table-top centrifuge (Eppendorf) for 15 min at maximum speed (14,000 rpm), and the supernatant was transferred to a new tube and used for β-gal activity assays and Western blotting.

To measure β-gal activity in tissues, T-Per protein extracts were diluted to 4 μg/μl by the addition of dilution buffer (5 mm NaPO_4_, 0.005% Tween 80, 150 mm NaCl, 0.1% BSA, pH 6.5). β-Gal activity was measured in duplicate by incubating 5 μl of T-Per tissue protein extract (containing 20 μg of protein/well) with 1.8 mm 4MU-GAL (Sigma-Aldrich, catalog no. M1633) in assay buffer (50 mm citrate, 125 mm NaCl, 0.5% Triton X-100, 0.1% BSA, pH 4.5) at 37 °C in a black 96-well plate (Corning). After 30 min, the reaction was stopped by the addition of 200 μl of stop buffer (0.5 m glycine, 0.3 M NaOH, pH 10.3) to each well, and fluorescence was measured on a FlexStation 3 multimode microplate reader (Molecular Devices, Sunnyvale, CA) with excitation wavelength at 355 nm and emission wavelength at 460 nm. The fluorogenic compound 4MU emits light when free in solution but not when covalently bound to a synthetic substrate.

Raw data were acquired using SoftMax Pro 6.3 (Molecular Devices) and transferred to Excel spreadsheets (Excel 2007, Microsoft Corp.) for analysis. The activity levels of the samples were calculated by extrapolating the amount of product (4MU) generated in the reaction from a seven-point standard curve that was prepared by adding a known amount of 4MU (Sigma-Aldrich, catalog no. M1381) into 20 μg/well of GM1 KO brain or liver protein extract used as background matrix. Activity levels are expressed as nmol of 4MU cleaved/mg of protein/h. The amount of active human β-gal in tissue homogenate samples from ICV treated GM1 KO mice was back-calculated from a seven-point standard curve that was prepared by adding a known amount of recombinant human β-gal (BioMarin Pharmaceutical Inc.) into 20 μg/well GM1 KO brain or liver protein extract used as matrix.

Analyzed data were entered into Prism 7 (GraphPad) to prepare the graphs used in the figures. Statistical analysis (one-way analysis of variance followed by Tukey's multiple-comparison test) was performed using Prism 7.

### Ganglioside and glycan analysis in mouse tissues

Tissue samples (homogenized in water) were analyzed for GM1 and GA1 levels with an Acquity UPLC attached to a Xevo TQ-S micro triple quadrupole mass spectrometer (Waters). Gangliosides were separated on an Acquity UPLC Glycan BEH Amide column (Waters). Samples were ionized by ESI in positive ion mode. The capillary voltage was set at 1.0 kV, the desolvation temperature was set at 500 °C, and the desolvation gas flow was 1000 liters/h. Two precursor-product ion transitions, one for the (d18:1/18:0) species and one for the (d18:1/20:0) species, were monitored for each of the five gangliosides. The sum of the two transitions for each ganglioside was used for quantitation. A standard reference curve containing all five gangliosides from 100 to 6.25 pg/μl was prepared with standards from Enzo Life Sciences, Inc. (Farmingdale, NY) in 95:5 methanol/glacial acetic acid (v/v).

The A2G2 (NA2) glycan (2000 pmol) was digested with Endo S (800 units) (New England Biolabs) in a total volume of 20 μl of 50 mm sodium phosphate, pH 7.5, for 18–24 h, followed by purification on a Sep-PAK C18 SPE cartridge (100 mg, 1 ml) (Waters), as per the manufacturer's instructions, and dried by centrifugal evaporation. Dried A2G2′ glycan standard was then labeled with [^13^C_6_]aniline (15 μl of 1 m NaCNBH_3_ in 70:30 DMSO/HOAc) at 37 °C for 18–24 h, and the remaining aniline was removed by centrifugal evaporation.

LC/MS analysis of samples for glycans was performed on an Acquity UPLC system equipped with a Glycan BEH Amide HILIC column (1.7 μm, 2.1 × 150 mm) (Waters) connected to a Thermo LTQ Orbitrap XL mass spectrometer (Thermo Scientific, Waltham, MA). Solvent A was 100 mm ammonium formate, pH 4.5, and solvent B was acetonitrile with an initial composition of 22% A, 78% B and a flow rate of 0.2 ml/min. The column temperature was kept at 60 °C. Prior to injection, an amount of aniline-labeled sample equal to 70 μg of protein was placed in an LC/MS sample vial along with 10 pmol of [^13^C_6_]aniline-labeled A2G2′ biomarker internal standard and dried by centrifugal evaporation. The samples were then dissolved in a solution of 22% A, 78% B. The labeled free glycans were eluted using a gradient profile of 22% A, 78% B to 37% A; 63% B over 65 min; 100% A, 0% B for 6 min; 100% A, 0% B to 22% A, 78% B in 5 min; and held there for 9 min. The LTQ Orbitrap XL was operated in the positive ion mode, and the sample was introduced by ESI. The capillary temperature was set at 250 °C with a capillary voltage of 2 kV. The sheath gas flow was set to 58, and the auxiliary gas flow was set to 9. Full scans were performed at a resolution of 60,000 and a range of 200–2250 *m*/*z*. The *N*-glycan A2G2′ metabolite was determined by ratiometric comparison of the [^12^C_6_]aniline-labeled endogenous A2G2′ ion abundance with that of the known molar amount of the internal standard spike as described previously.^4^ A full characterization of the glycan substrates accumulating in GM1 gangliosidosis mice has recently been reported (4).

## Supplementary Material

Supporting Information

## Footnotes

4 GlobeNewswire, May 16, 2019: Axovant Announces Dosing of First Patient in Clinical Program for AXO-AAV-GM1, a Novel Gene Therapy for GM1 Gangliosidosis, Axovant, Ltd., London, UK.

## Footnotes

3 The abbreviations used are:

GM1: monosialotetrahexosylganglioside
GA1: asialo-GM1
ERT: enzyme replacement therapy
CHO: Chinese hamster ovary
MPR: mannose 6-phosphate receptor
CI-MPR: cation-independent MPR
ER: endoplasmic reticulum
SV-AUC: sedimentation velocity analytical ultracentrifugation
SE-AUC: sedimentation equilibrium AUC
DSF: differential scanning fluorimetery
CZE: capillary zone electrophoresis
HDX: hydrogen–deuterium exchange mass spectrometry
CSF: cerebrospinal fluid
aCSF: artificial CSF
HILIC: hydrophilic interaction chromatography
APTS: 8-aminopyrene-1,3,6-trisulfonic acid
KS: keratan sulfate
CNS: central nervous system
ICV: intracerebroventricular or intracerebroventricularly
AAV: adeno-associated virus
rhβ-gal: recombinant human β-gal
PoC: proof-of-concept
IHC: immunohistochemistry
X-gal: 5-bromo-4-chloro-3-indolyl β-d-galactoside
CMV: cytomegalovirus
PDI: protein-disulfide isomerase
CHOP: C/EBP-homologous transcription factor
PPCA: protective protein cathepsin A
Neu1: neuraminidase 1
4MU: 4-methylumbelliferone
4MU-GAL: 4-methylumbelliferyl-β-d-galactopyranoside fluorogenic substrate
FA: formic acid
AF488: Alexa Fluor 488
MOI: multiplicity of infection
KO: knockout
PRM: parallel reaction monitoring
OFB: olfactory bulb
CBX: cerebral cortex
HS: hypothalamus and septum
CB: cerebellum
MB: mid-brain (containing pons and medulla)
M6P: mannose 6-phosphate
IF: immunofluorescence.
