# Supplementary material for: Intracerebroventricular enzyme replacement therapy with β-galactosidase reverses brain pathologies due to GM1 gangliosidosis in mice
Source: J Biol Chem. 2019 Sep 3;295(39):13532–55. doi: 10.1074/jbc.RA119.009811 (PMC7521651; doi:10.1074/jbc.RA119.009811)
Supplement: Supporting Information [file supp_295_39_13532__index.html]

Intracerebroventricular enzyme replacement therapy with β-galactosidase reverses brain pathologies due to GM1 gangliosidosis in mice — Evaluation of β-gal ERT and gene therapy for GM1 gangliosidosis — Supporting Information 

# Intracerebroventricular enzyme replacement therapy with β-galactosidase reverses brain pathologies due to GM1 gangliosidosis in mice

## Supporting Information

- Supplemental figures-S1-S5
