# Supplementary material for: Intracerebroventricular enzyme replacement therapy with β-galactosidase reverses brain pathologies due to GM1 gangliosidosis in mice
Source: J Biol Chem. 2019 Sep 3;295(39):13532–55. doi: 10.1074/jbc.RA119.009811 (PMC7521651; doi:10.1074/jbc.RA119.009811)
Supplement: Supporting Information [file supp_RA119.009811_Supplemental-Figures-S1-S5.pdf]

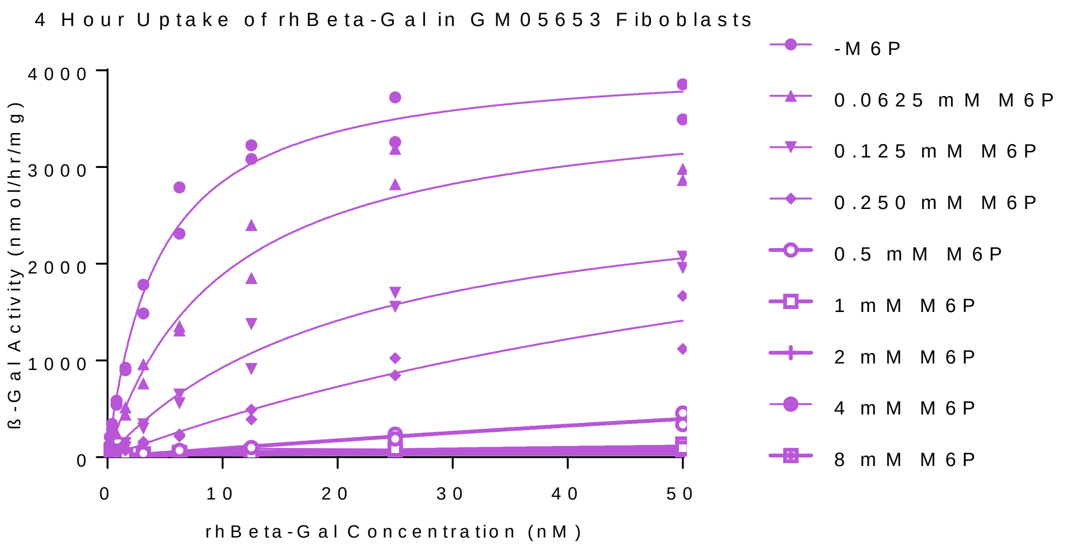

Figure S1: Supporting information for Fig 1B of manuscript, showing that the majority of rhBeta-Gal cellular uptake into GM05653 GM1 gangliosidosis patient fibroblasts can be inhibited with doses of M6P that are equal to or greater than 1 mM.

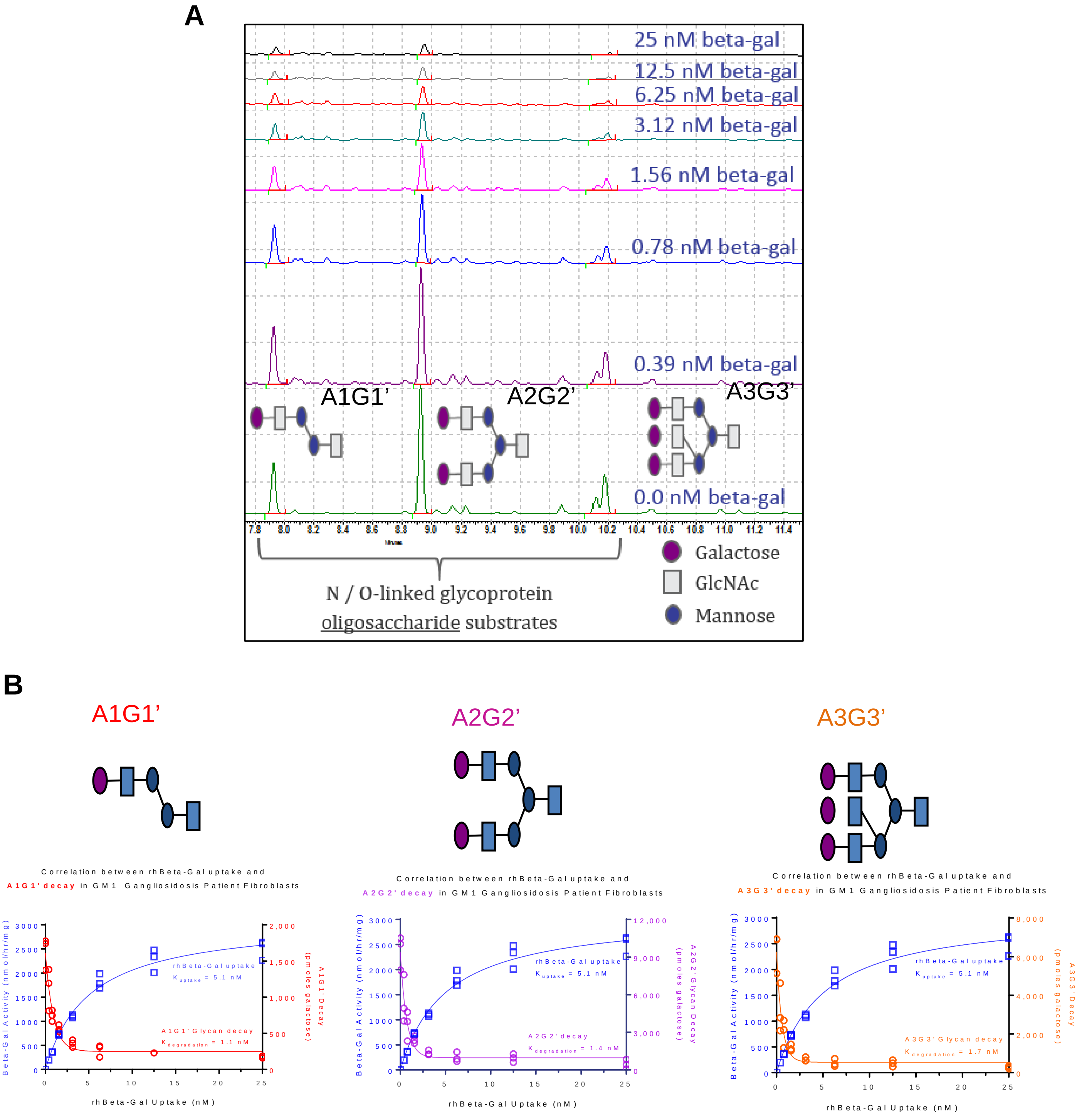

Fig S2: Supporting information for Fig 1D and 1E of manuscript.

A. Representative Capillary Zone Electrophoresis (CZE) analysis of APTS-labeled glycans from GM1 gangliosidosis fibroblast lysates shows the accumulation of 3 major glycan species (A1G1', A2G2', A3G3'), with their predicted structures indicated. rhBeta-Gal (beta-gal) uptake for 4 hours was performed in triplicate cultures of GM05653 cells, followed by a 6 hour chase results in dose-dependent clearance of the glycan substrates (see above). ***B.*** Structure of A1G1', A2G2', A3G3' and their individual clearance rates in GM1 gangliosidosis patient fibroblasts following rhBeta-Gal cellular uptake. The quantified amount of each major glycan detected by CZE in A is plotted against the level of Beta-Gal activity detected following cellular uptake of rhBeta-Gal. Very low nM concentrations of rhBeta-Gal uptake are sufficient to clear all three major glycan species ($K_{degradation}$=1.1 nM, 1.4 nM and 1.7 nM for A1G1', A2G2' and A3G3', respectively).

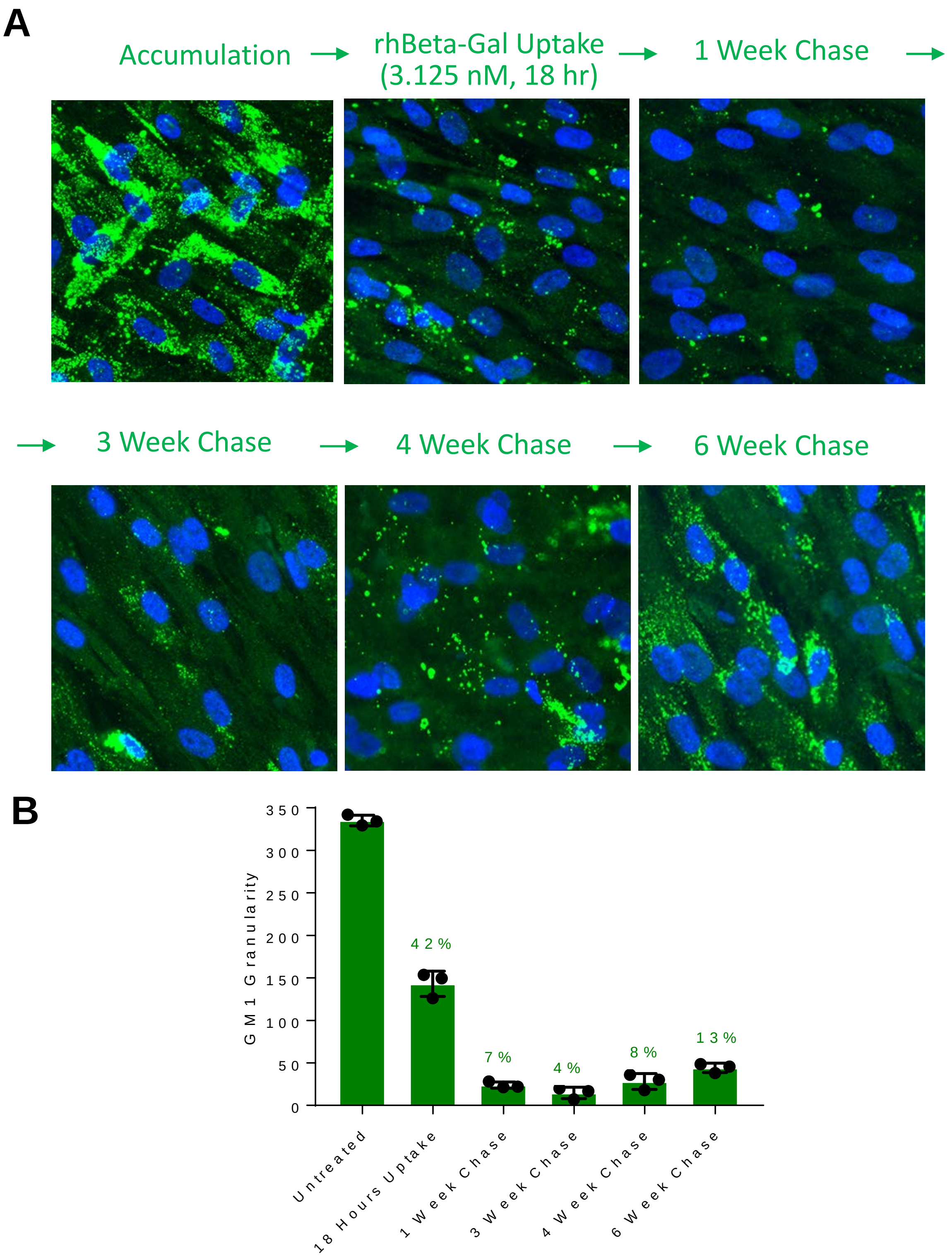

Fig S3: Supporting information for Fig 1G of manuscript, with high-content imaging being used to detect Beta-Gal substrate levels in GM1 gangliosidosis patient fibroblasts using a commercially available GM1 polyclonal antibody. Cells were grown for ten days after reaching confluence to permit substrate accumulation, at which time cells were treated with rhBeta-Gal for 18 hours. The uptake medium was removed and cells were rinsed several times, then chased for up to six weeks with twice weekly feeding but without passaging***. A,*** Representative high content images of each treatment group, acquired at 20X magnification. **B.** Quantified granularity from four fields in three independent cultures of cells for each treatment as described in the Materials and Methods.

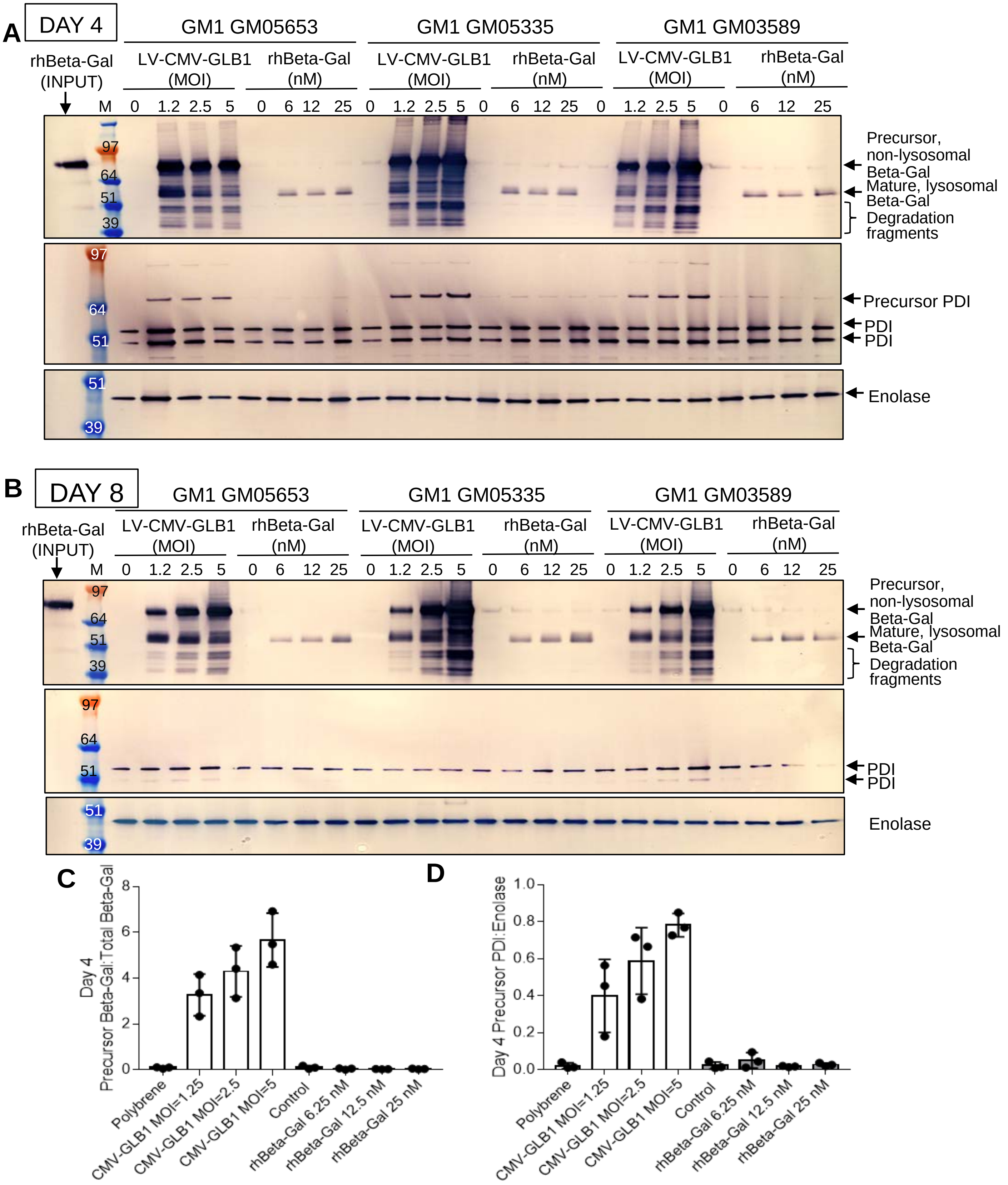

Fig S4: Supporting information for Figure 8, showing that chronic lentiviral-mediated GLB1 over-expression for 4 days in the three GM1 gangliosidosis patient fibroblast lines coincides with an increase in precursor Beta-Gal and precursor PDI protein levels (panel A, quantification in panel C and D). In contrast, after 8 days of chronic GLB1 over-expression precursor PDI is no longer detected in the gene therapy treated cells (panel B), suggesting that the increased precursor PDI protein levels detected at Day 4 is an early and transient unfolded protein response to high levels of GLB1 over-expression.

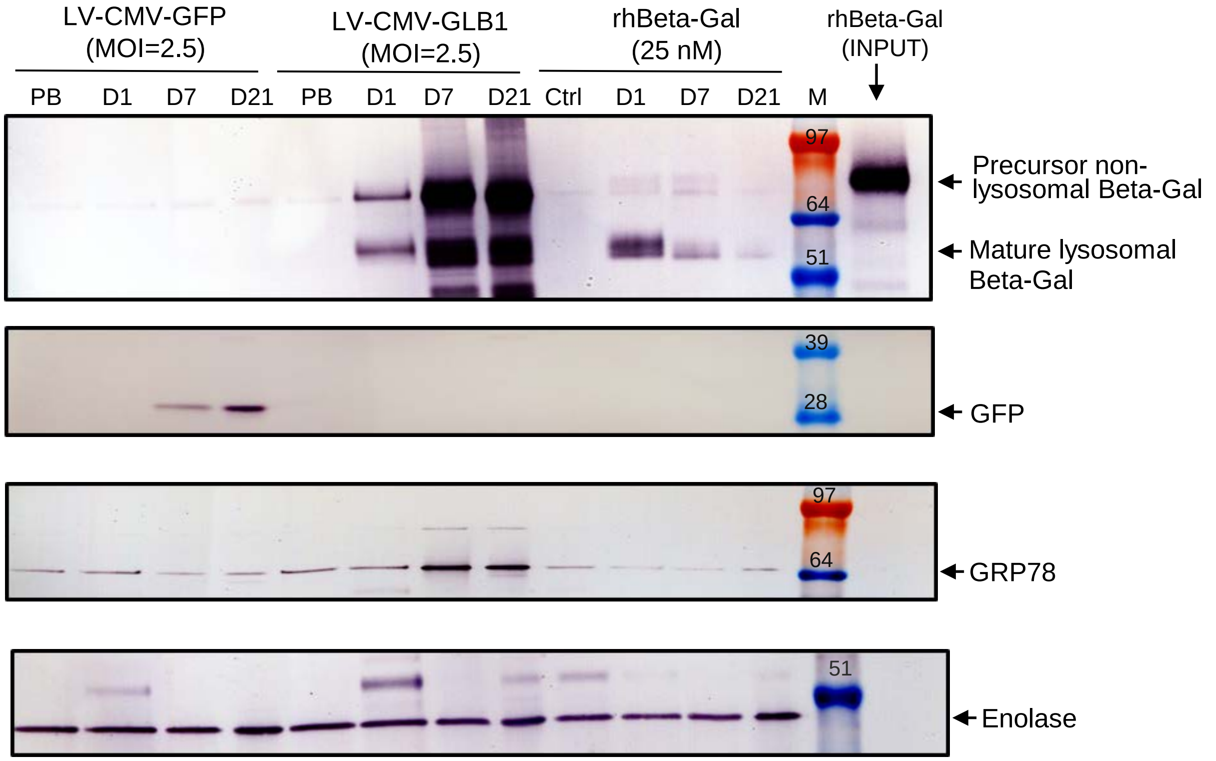

Fig S5: Supporting information for Fig 8 of manuscript, showing that lentiviral-mediated GLB1 over-expression in GM1 gangliosidosis patient fibroblasts coincides with a time-dependent increase in the detection of precursor Beta-Gal protein, and Grp78 protein levels. Western blots of cell lysates prepared from GM05653 GM1 gangliosidosis patient fibroblasts 1, 7 or 21 days after being transduced with LV-CMV-GFP control virus or LV-CMV-GLB1 virus for 24 hours in the presence of polybrene (PB), or 1, 7 or 21 days after cellular uptake with purified rhBeta-Gal (24 hour enzyme uptake).
